# Exploring the Intersection of Artificial Intelligence and Clinical Healthcare: A Multidisciplinary Review

**DOI:** 10.3390/diagnostics13121995

**Published:** 2023-06-07

**Authors:** Celina Silvia Stafie, Irina-Georgeta Sufaru, Cristina Mihaela Ghiciuc, Ingrid-Ioana Stafie, Eduard-Constantin Sufaru, Sorina Mihaela Solomon, Monica Hancianu

**Affiliations:** 1 Department of Preventive Medicine and Interdisciplinarity, Grigore T. Popa University of Medicine and Pharmacy Iasi, Universitatii Street 16, 700115 Iasi, Romania; celina.stafie@umfiasi.ro; 2 Department of Periodontology, Grigore T. Popa University of Medicine and Pharmacy Iasi, Universitatii Street 16, 700115 Iasi, Romania; 3 Department of Morpho-Functional Sciences II—Pharmacology and Clinical Pharmacology, Grigore T. Popa University of Medicine and Pharmacy Iasi, Universitatii Street 16, 700115 Iasi, Romania; 4 Endocrinology Residency Program, Sf. Spiridon Clinical Emergency Hospital, Independentei 1, 700111 Iasi, Romania; 5 Technology Reply Romania, Ceasornicului 17, 014111 Bucharest, Romania; 6 Pharmacognosy-Phytotherapy, Grigore T. Popa University of Medicine and Pharmacy Iasi, Universitatii Street 16, 700115 Iasi, Romania

**Keywords:** allergology, artificial intelligence, cardiology, dentistry, diagnosis, immunology, machine learning, prediction, treatment

## Abstract

Artificial intelligence (AI) plays a more and more important role in our everyday life due to the advantages that it brings when used, such as 24/7 availability, a very low percentage of errors, ability to provide real time insights, or performing a fast analysis. AI is increasingly being used in clinical medical and dental healthcare analyses, with valuable applications, which include disease diagnosis, risk assessment, treatment planning, and drug discovery. This paper presents a narrative literature review of AI use in healthcare from a multi-disciplinary perspective, specifically in the cardiology, allergology, endocrinology, and dental fields. The paper highlights data from recent research and development efforts in AI for healthcare, as well as challenges and limitations associated with AI implementation, such as data privacy and security considerations, along with ethical and legal concerns. The regulation of responsible design, development, and use of AI in healthcare is still in early stages due to the rapid evolution of the field. However, it is our duty to carefully consider the ethical implications of implementing AI and to respond appropriately. With the potential to reshape healthcare delivery and enhance patient outcomes, AI systems continue to reveal their capabilities.

## 1. Introduction

### 1.1. What Is AI?

Artificial intelligence (AI) is the simulation of human intelligence processes by machines or computer systems. Natural language processing, speech recognition, expert systems, or machine vision are common applications of AI. In simple words, the AI field combines computer science and good-quality, vetted, datasets for the purpose of solving a given problem. It also encompasses sub-fields of machine learning and deep learning, which are frequently mentioned in conjunction with artificial intelligence [1]. These disciplines are comprised of AI algorithms that seek to create expert systems, which make predictions or classifications based on input data. Over the years, artificial intelligence has gone through many cycles of hype, but even to skeptics, the ChatGPT release of OpenAI seems to mark a turning point [2]. The last time generative AI loomed this large, the breakthroughs were in computer vision [3], but now the leap forward is in natural language processing. Moreover, it extends beyond language; generative models possess the ability to acquire the grammar of software code, molecules, natural images, and diverse types of data [4].

### 1.2. How Does It Work?

In general, AI systems operate by consuming substantial amounts of labeled data for training purposes [5]. They analyze this data to identify correlation patterns, which are then utilized to make predictions about future events or states. For instance, a chatbot trained on text examples can learn to generate realistic conversations with individuals, while an image recognition tool can learn to identify and describe objects in images by studying millions of examples [2]. The advancement of generative AI techniques has enabled the creation of lifelike text, images, music, and other media.

Currently, AI plays prominent roles in medical settings, primarily in clinical decision support and imaging analyses. Clinical decision support tools assist in making informed decisions related to treatments, medications, mental health, and other patient needs by swiftly providing relevant information or research [6]. In the field of medical imaging, AI tools are utilized to analyze various types of scans such as computer tomography (CT), X-rays, magnetic resonance imaging (MRI), and others [7]. They help identify lesions or other findings that may be overlooked by human observers.

### 1.3. Methodology

This narrative review provides an insight into main branches of artificial intelligence and different applications in cardiology, allergology, immunology, endocrinology, and dentistry, as well as an approach on advantages and limitations of AI in medicine. The search was conducted on MEDLINE/PubMed, Web of Science, and Scopus, and the last search was performed on 25 March 2023. Typical keywords involved ‘‘artificial intelligence’’ ∧ (‘‘healthcare’’ ∨ ‘‘medicine’’ ∨ ‘‘deep learning’’ ∨ ‘‘machine learning’’), as well as various combinations. A total of 151 studies published in the English language were gathered as the base for the initial literature corpus, with 88% of them being published in the past 5 years.

## 2. Artificial Intelligence Branches Explained

It is important to understand the different concepts in artificial intelligence that help solve real-world problems—the reason why we will go over the main branches of AI, such as:

### 2.1. Machine Learning

Machine learning (ML) is the ability of machines to automatically learn from data and algorithms. It improves the performance using past experiences [8].

The process starts with historical data collection, such as instructions and direct experience, so that logical models can be built for future inference. Output accuracy depends on data size—a larger amount of data will build a better model, which in turn increases its accuracy [9].

### 2.2. Computer Vision

This branch of AI aims to develop techniques that assist computers in seeing and understanding digital images and videos [3]. Applying machine learning models to images allows computers to identify objects, faces, people, animals, and more. Algorithmic models help computers teach themselves about visual data contexts, and with enough data fed through a model, computers can teach themselves to distinguish one image from another [10].

### 2.3. Fuzzy Logic

Fuzzy logic is a technique that helps to solve issues or statements that can either be true or false. This method copies human decisions by considering all existing possibilities between digital values of ‘yes’ and ‘no’. Put simply, it measures the degree to which a hypothesis is correct [11].

This branch of artificial intelligence is used to reason about uncertain topics. It is a convenient and flexible way of implementing machine learning techniques and copying human thought logically [12].

The architecture of fuzzy logic is composed of four parts:

▪ Rule base: Has all the rules and if–then conditions.
▪ Fuzzification: Helps to convert inputs.
▪ Inference engine: Determines the degree of match between rules and fuzzy inputs.
▪ Defuzzification: Converts fuzzy sets into crips values.

### 2.4. Expert Systems

An expert system is a program specializing in a singular task, similar to a human expert. These systems are mainly designed to solve intricate problems with human-like decision-making capabilities [13]. They use a set of rules, called inference rules, that a knowledge base fed by data defines for them. By using if–then logical notions, they can solve complex issues.

### 2.5. Robotics

Robots are programmed machines that can automatically carry out complex series of actions. People control them with external devices, or their control systems can be embedded within themselves. Surgical-assistance robots are designed to enhance existing surgical treatments, including minimally invasive surgeries and orthopedic surgeries [14]. These types of robots can be used to perform bariatric surgery and knee and hip replacement procedures, among other surgeries.

### 2.6. Neural Networks/Deep Learning

Neural networks are also known as artificial neural networks (ANNs) or simulated neural networks (SNNs). At the heart of deep learning algorithms, neural networks are inspired by the human brain, and they copy how biological neurons signal to each other. Neural networks need training data to both learn and improve accuracy [15]. Deep learning models can recognize complex patterns in pictures, text, sounds, and other data to produce accurate insights and predictions [16]. It can be visualized as a complex network of interconnected nodes that process data in layers, where each layer extracts increasingly complex features from the input data.

### 2.7. Natural Language Processing

Natural language processing (NLP) allows computers to understand both text and spoken words in a similar manner to humans. Combining machine learning, linguistics, and deep learning models, computers can process human language in voice or text data to understand the full meaning, intent, and sentiment [17]. In speech recognition or speech-to-text, for example, voice data are reliably converted to text data. This can be challenging as people speak with varied intonations, emphasis, and accents [18]. Programmers have to teach computers natural language-driven applications so they can understand and recognize data from the beginning.

## 3. Artificial Intelligence in Medicine

### 3.1. A Brief History of AI

AI has known a dramatic evolution in the past seven decades. Initially, the emphasis of AI was directed towards creating machines able to make interferences or decisions that were typically limited to human capabilities. This pursuit led to the introduction of the first industrial robot arm, Unimate, which was capable of following step-by-step commands. Unimate became part of General Motors’ assembly line in 1961 and effectively carried out automated die casting [19]. In 1964, Eliza was developed, using natural language processing, to mimic human conversation; Eliza is considered the framework for the chatbots of today [20]. A significant advancement took place in 1966, with the creation of Shakey at the Stanford Research Institute. Shakey, “the first electronic person”, was able to process complex instructions and execute the appropriate action [21]. The emergence of clinical informatic databases and medical record systems during this era played a foundational role in shaping the future advancements of AI in medicine [22].

A pioneering example showcasing the potential of applying AI to the field of medicine is the creation of a consultation program for glaucoma, known as the CASNET model, at Rutgers University. This model was presented at the Academy of Ophthalmology meeting in Las Vegas, Nevada, in 1976 [20]. CASNET incorporated a causal-association network and comprised three distinct programs: model building, consultation, and a collaboratively built and maintained database.

MYCIN was developed in the early 1970s, an AI system with a knowledge base of 600 rules, which could provide a list of pathogens and antibiotic recommendations, adjusted for the body weight of the patient [20]. The University of Massachusetts introduced DXplain in 1986. DXplain utilizes inputted symptoms to generate a comprehensive list of potential diagnoses, based then on 500 diseases. Its database has since expanded to encompass over 2400 diseases by the year 2015.

Deep Learning (DL) represents a significant leap forward in the field of AI in medicine. In contrast to machine learning, which relies on a predetermined set of features and requires human input, DL possesses the capability to autonomously classify data. While DL was initially explored in the 1950s, its application in medicine faced challenges related to “overfitting”. Overfitting arises when ML becomes excessively focused on a specific dataset, rendering it less effective in processing new datasets. This limitation was primarily attributed to an inadequate computing capacity and scarcity of training data [23]. However, in the 2000s, these obstacles were overcome due to the availability of larger datasets and significant advancements in computing power.

IBM built a system similar to DXplain, Watson, in 2007; Watson is able to analyze unstructured content and find probable answers, based on various searches and natural language processing (NLP) [24]. This particular system helped in identifying the binding of RNA proteins [25]. Based on NLP, chatbots such as Pharmabot [26] and Mandy [27] were developed to assist patients in gaining a clearer understanding of their medications.

### 3.2. Artificial Intelligence Algorithms in Medicine

AI algorithms appear to be magical, but they are simply mathematical functions that describe input data and map them to outputs. The input data in healthcare includes structured elements such as diagnostic codes, vital sign fields, and demographic fields, as well as unstructured data such as text and radiologic images. Input data should be selected based on features relevant to the desired prediction task. A variety of models must be applied to test for the best performance since inputs can be highly variable with no clear interrelationships between them [28].

The four fundamental tasks listed under the term of competence, i.e., diagnosing, estimating a prognosis, identifying causes of diseases, and selecting treatments, must be gained from patients prior to a diagnosis. It was not until the twentieth century that data were collected beyond medical histories and physical examinations conducted in the privacy of medical offices. Following this, data collection underwent an industrial revolution, characterized by machine use and division of labor [29]. As medical decisions become more complex due to the proliferation of data sources and the need to involve multiple specialists, physicians have shifted from making individual decisions in the privacy of their offices to making collective decisions in multidisciplinary meetings.

After the first step of collecting data from their patients, the second step for physicians is to use their clinical reasoning to make medical decisions. From an informatics point of view, clinical reasoning is data processing; data can be processed by algorithms, and algorithms are currently able to deliver a diagnostic probability, a prognostic estimation, or the selection of a treatment. As data collection and data processing/AI have progressed, the number of actors involved in patient care has multiplied, and these actors are no longer limited to humans but also software that makes medical decisions. In order to maintain their “competence” dimension, physicians must retain control over these new technologies [30].

AI tools fit along a continuum between fully human and fully computer-driven on the concept of a human–computer collaboration spectrum. This view emphasizes levels of analytic complexity that provide a framework for clarifying forms of machine learning. In contrast, a deep learning model can automatically classify inputs without much human intervention, unlike a classic statistical model.

**Figure 1:**
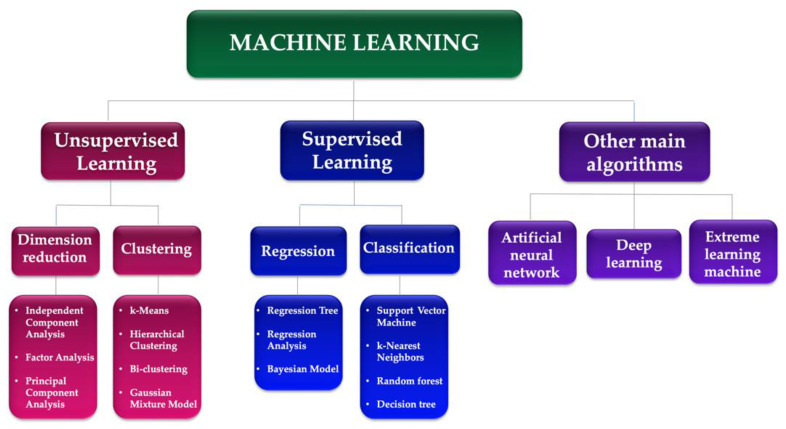
Machine learning algorithms.

Figure 1 synthesizes the main methods and algorithms of machine learning used in medicine:

(1) Supervised learning: These models are trained on labeled data to learn patterns and make predictions. They are widely used in medical image analyses, such as identifying cancer cells in pathology images or detecting lung nodules in CT scans [7]. Some examples of supervised learning models used in medicine include convolutional neural networks (CNNs), deep neural networks (DNNs), and random forests.
(2) Unsupervised learning models: These models are used to identify patterns and relationships in unlabeled data. They are used in medical data clustering, anomaly detection, and feature extraction. Some examples of unsupervised learning models used in medicine include k-means clustering, a principal component analysis (PCA), and autoencoders [31].
(3) Reinforcement learning models: These models are used to learn from trial-and-error interactions with an environment. They can be used in medical decision making, such as personalized treatment planning and drug dosage optimization [32]. Examples of such models include Q-learning, policy gradient methods, and actor–critic models.
(4) Hybrid models: These models combine multiple types of AI models to leverage their strengths and overcome their weaknesses. For example, a hybrid model could use a CNN to identify features in medical images followed by an unsupervised learning algorithm to cluster the features and identify subtypes of cancer [33].

The main AI systems used in medicine are synthesized in Table 1.

## 4. AI in Cardiology

AI found its applications in cardiology in several domains, such as imaging, electrophysiology, and heart failure prediction, as well as preventive and interventional cardiology. AI models can be used for an electrocardiogram (ECG) analysis. Deep learning models have been shown to accurately identify cardiac arrhythmias, including atrial fibrillation (AF), and ventricular tachycardia, from ECG recordings [50]. A growing number of people are likely to develop AF within the next three decades [51], making early diagnoses and management critical in primary care. By screening for undetected AF in primary care populations, ML models may enable early anticoagulation and reduce the subsequent disease burden. For the diagnosis of AF, ML models are trained on clinical data from electronic patient records (EPRs) or ECGs [52]. By using the former approach, clinicians can assist patients with screening based on age, previous cardiovascular disease, and body mass index. A ML model based on an ECG waveform analysis has demonstrated high accuracy. On the basis of 18,000 ECG signals, a deep learning system can diagnose atrial fibrillation with an accuracy of 98.27% [53]. Studies have demonstrated that many general practitioners are incapable of accurately detecting and diagnosing AF based on ECGs [9]. ECGs may be useful in identifying high-risk patients, subsequently resulting in a combined approach pathway. Any non-trivial traces can then be identified or flagged for specialist intervention.

ML models have also been used to automatically identify and quantify cardiac function parameters, such as the ejection fraction, without the need for manual measurements [16]. AI can also assist in the prediction of the cardiovascular disease (CVD) risk. A study by Weiss et al. [54] demonstrated that a deep learning model can predict the 10-year risk of cardiovascular disease more accurately than traditional risk calculators, based on a single chest X-ray. Additionally, AI can help identify patients who may benefit from preventive interventions or close monitoring, potentially reducing the risk of adverse cardiovascular events. The main outcomes of AI use in cardiology are presented in Table 2.

AI has also been applied in the prediction of patient outcomes. AI algorithms can analyze specific parameters to predict the likelihood of a patient developing certain complications, such as heart failure or a stroke. Hamatani et al. [55] used a ML model based on a random forest algorithm to assess the heart failure hospitalization in patients with atrial fibrillation. The proposed model exerted a higher prediction performance than the Framingham risk model [55]. In a systematic review, Kee et al. [56] observed that a neural network was able to predict the risk of CVD in type 2 diabetes patients, with 76.6% precision and 88.06% sensitivity [56].

**Table 2:** Overview of AI use in Cardiology.

Target	Type of Algorithm	Data Sample	Results	Study
Signal processing				
Detection of VF and VT (shockable rhythms) to improve shock advice algorithms in automated external defibrillators	Convolutional neural network as a feature extractor and boosting classifier	1135 shockable segments and 5185 non-shockable segments from 57 records in public databases	Accuracy 99.3%, sensitivity 97.1%, specificity 99.4%	Nguyen et al., (2018) [57]
Automated detection of AF based on PPG and accelerometer recordings of smartwatches	Deep neural network with heuristic pre-training	Heart rate and step count data obtained using the Cardiogram mobile application on Apple Watches from 9759 Health eHeart Study participants	Sensitivity 98.0%, specificity 90.2%, C-statistic 0.97	Tison et al., (2018) [58]
Binary classification ofcardiovascular abnormality using time–frequency features of cardio-mechanical signals, namely, SCG and GCG signals	Decision tree and SVM methods with features generated by a continuous wavelet transform	Experimental measurements from 12 patients with cardiovascular diseases and 12 healthy subjects	Accuracy > 94%, with the best performance of SVM applied to GCG features (99.5%)	Yang et al., (2018) [59]
Automated detection of AF based on Apple Watch Series 2 or 3 with KardioBand (AliveCor)	SmartRhythm 2.0, a convolutional neural network	Data of the heart rate, activity level, and ECGs from 7500 AliveCor users (training), and data from 24 patients with an insertable cardiac monitor and history of paroxysmal AF (validation)	Episode sensitivity 97.5%, duration sensitivity 97.7%, patient sensitivity 83.3% overall and 100% during time worn	Wasserlauf et al., (2019) [60]
Identify LV territory of regional wall motion abnormality on parasternal short-axis views	Convolutional neural networks (supervised)	In total, 400 patients (1200 short-axis echo videos) who had undergone a coronary angiography and echo	Area under the receiver operating characteristic curve for detection of regional wall motion abnormalities: 0.90–0.97	Kusunose et al., (2019) [61]
Identification of asymptomatic LV dysfunction based on an ECG	Convolutional neural network using the Keras framework with a Tensorflow (Google) backend and Python	ECG–TTE pairs: 35,970 (training), 8989 (internal validation), 52,870 (testing)	Accuracy 85.7%, sensitivity 86.3%, specificity 85.7%, C-statistic 0.93	Attia et al., (2019) [50]
Image processing				
Rapid and reproducible measurement of LV volumes, EF, and average biplane LS on ECG	Convolutional neural networks	Four- and two-chamber ECG views from 255 patients in sinus rhythm	Feasibility 98%, good agreements with the reference for automated EF and LS, with no variability	Knackstedt et al., (2015) [62]
Decreasing the computational demand of the FFR calculation by developing a ML-based model as an alternative to computational fluid dynamics approaches	Deep neural network	In total, 125 lesions in 87 patient-specific anatomic models generated from CT data using image segmentation	Excellent correlation (0.9994; *p* < 0.001) and no systematic bias in the Bland–Altman analysis: FFR 0.80 was predicted with sensitivity 81.6%, specificity 83.9%, accuracy 83.2%	Itu et al., (2016) [63]
Automated ECG interpretation, including view identification, segmentation of cardiac chambers across five commonviews, quantification of structures and function, and disease detection	Convolutional neural networks	In total, 14,035 echocardiograms spanning a 10-year period	Identification of views in >95%, median absolute deviation of 15–17% for structure and <10% for function, detection of hypertrophic cardiomyopathy, cardiac amyloidosis, and pulmonary disease with C-statistics of 0.93, 0.87, and 0.85, respectively	Zhang et al., (2018) [64]
Measurement of RV and LV volume and function in MRI images for a range of clinical indications and pathologies	Deep neural network	In total, 200 non-congenital clinical cardiac MRI examinations	Strong correlations for LV (>0.94) and RV (>0.92) volumes	Retson et al., (2020) [65]
Detection of subclinical AF	Convolutional neural networks	Training set of 454,789 images and testing on 130,801 images	AUC 0.90, sensitivity 82.3%, specificity 83.4%, accuracy 83.3%	Alzubaidi et al., (2021) [66]
Clinical risk stratification				
Identification of HF cases from both structured and unstructured EMRs	Random forest models	In total, 2,139,299 notes in the Maine Health Information Exchange EMR database from 1 July 2012 to 30 June 2014	Positive predictive value of 91.4%	Wang et al., (2015) [67]
Development of CHIEF to automatically extract LV function mentions and values, congestive HF medications, and documented reasons for a patient not receiving these medications	Combination of rules, dictionaries, and ML methods	Various clinical notes from 1083 Veterans Health Administration patients	High recall and precision for HF medications and EF (>0.960), while only reaching fair recall and precision for reasons for not prescribing HF medications (<0.400)	Meystre et al., (2017) [68]
Risk prediction model of incident essential hypertension within the following year	Feature selection and generation of an ensemble of classification trees with the use of XGBoost	Data from individual patient electronic health records as part of the Health Information Exchange data set of Maine	C-statistics of 0.917 in the retrospective cohort and 0.870 in the prospective cohort	Ye et al., (2018) [69]
Predict survival following a routine echo using clinical and structured echo report data	Nonlinear random forest classifier (supervised)	In total, 171,519 patients (331,317 echo studies) using 90 clinical variables, LVEF, and 57 echo measurements. Labels were from clinical data and reported echo measurements	Area under the receiver operating characteristic curve: 1-year mortality, 0.85 5-year mortality, 0.89	Samad et al., (2019) [70]
Predict in-hospital mortality following an echo in patients admitted with heart disease using echo report data	Deep neural network (supervised)	In total, 25,776 in-patients admitted with heart disease based on ICD-10 codes. Labels were from clinical data and reported echo measurements	Area under the receiver operating characteristic curve: Overall, 0.90 Coronary heart disease subgroup, 0.96 Heart failure subgroup, 0.91 Area under the precision–recall curve, 0.28	Kwon et al., (2019) [71]
Prediction of CAD on CTA	Boosted ensemble algorithm	Clinical, CTA (CACS) in 13,054 subjects	AUC 0.881	Lu et al., (2022) [72]

AF, atrial fibrillation; CHIEF, Congestive Heart Failure Treatment Performance Measure Information Extraction Framework; CT, computed tomography; ECG, electrocardiography; EF, ejection fraction; EMR, electronic medical record; FFR, fractional flow reserve; GCG, gyrocardiography; HF, heart failure; LS, longitudinal strain; LV, left ventricular; ML, machine learning; MRI, magnetic resonance imaging; PPG, photoplethysmography; RV, right ventricular; SCG, seismocardiography; SVM, support vector machine; TTE, transthoracic echocardiography; VF, ventricular fibrillation; VT, ventricular tachycardia.

A self-taught ML model was found to be better at predicting the risk of death in CVD patients than other models designed by cardiovascular experts [73]. Samad et al. [70] used echocardiographic and clinical parameters on a supervised learning model in order to obtain the survival prediction, compared to other risk scores and logistic regression models.

Personalized treatment is another area where AI can be useful in cardiology. AI algorithms can analyze patient data to identify the most effective treatment options for individual patients based on their unique characteristics. Chi et al. [74] used a machine learning personalized statin treatment plan to assess the available statin plans and to identify the optimal treatment plan in order to prevent or minimize statin patient discontinuation.

## 5. AI Implications in Immunology, Allergology, and COVID-19

The potential clinical applications of AI in allergies and immunology have a wide range, from a common disease diagnosis (food allergy, asthma, and drug allergy) to diseases with a delayed diagnosis, which fail to be very obvious from the beginning to the general practitioners and pediatricians, thereby endangering newborns’ lives, including the inborn errors of immunity. Other potential clinical applications include the assessment and prediction of adverse reactions to drugs and vaccines—to the pandemic pathology and the post-vaccination immune response to coronavirus disease 2019 (COVID-19) and non-COVID-19, and to the multidimensional data reduction in the electronic field, the health records, and the immunological datasets.

One area where AI has been applied in allergology is in the diagnosis of allergic diseases. AI algorithms can analyze patient data, such as medical history, allergy testing results, and environmental exposure data, to identify patterns and associations that may be indicative of allergic diseases. Yang et al. developed an ensemble neural network chain model with pre-training on rhinitis multi-label classification. Malizia et al. [75,76] established a machine learning model that, based on nasal cytology and skin prick test results, could identify allergic rhinitis phenotypes in children. Nevertheless, the authors acknowledge that cytologic endotypes over time may limit the efficiency of such a model [75]. Bhardwaj et al. [77] successfully trained and tested six ML models to classify allergic and non-allergic asthma.

AI methods have also been used in the prediction of allergic diseases and complications. Research conducted by van Breugel focused on a multi-omics model, which could accurately perform a methylation-based allergy diagnosis [78]. Therefore, ML models are able to go beyond a simple analysis of one domain and to integrate multi-omics layers [79]. AI algorithms can analyze patient data to predict the likelihood of a patient developing severe allergic reactions, such as anaphylaxis [80].

Another important aspect that could gain a major benefit from AI and ML is the discovery of drug allergies by establishing a risk profile for patients at risk of developing a drug allergy. The most common example is the beta-lactam amoxicillin and clavulanic acid combination, responsible in the last decade for late and immediate hypersensitivity reactions [81].

AI has been applied in the development of personalized treatment plans. AI algorithms can identify, based on patient data, the most effective individual treatment options [82].

**Figure 2:**
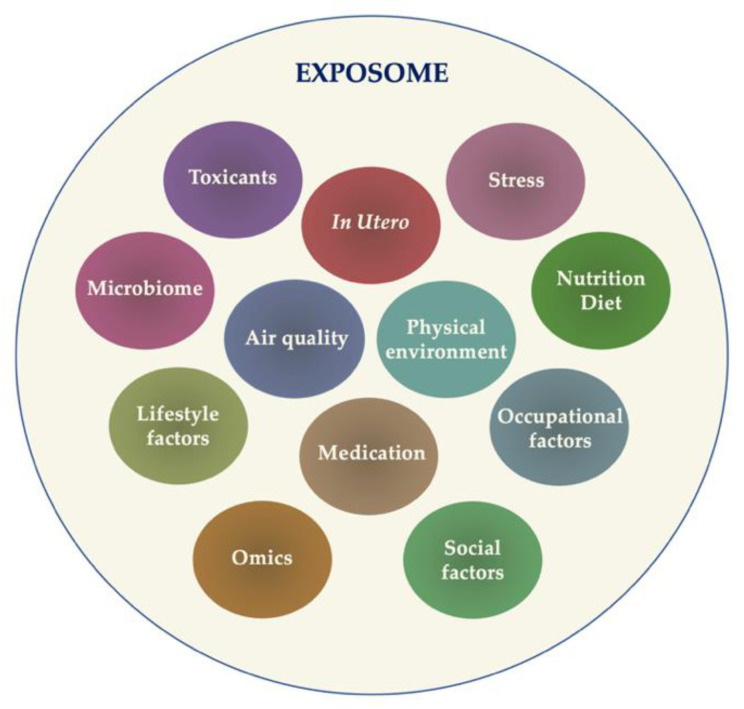
The sum of complex exposures (exposome).

ML endeavors to accomplish precision medicine in allergology by characterizing allergic endotypes, exploring relationships in allergic multimorbidity, contextualizing the impact of an exposome, and intervening in biological processes to enhance health and treat individual diseases. Exposure represents a critical factor in the allergic disease physiopathology, a high complexity factor mainly due to the possibility of multiple exposures that can occur simultaneously. The concept of an “exposome” has been introduced, a term that encloses “all exposures from conception onwards” [83]—a complex puzzle that can be put together by ML algorithms (Figure 2). Nevertheless, the beneficial role of ML in the exposome investigation is closely related to the quality of analyzed data.

Contemporary ML techniques employ embeddings to transform high-dimensional feature spaces into efficient representations. These approaches often leverage modern DL methods such as convolutional neural networks [84]. While these approaches demonstrate high prediction accuracy, it is essential to acknowledge that the patterns identified by these methods may be merely correlative, lacking direct associations with the underlying molecular mechanism. Nevertheless, they remain valuable as biomarkers in clinical assessments.

A convolutional neural network was used to accurately identify and count airborne pollen, to distinguish between the low-allergenic *Urtica* species and severely allergenic *Parietaria* species [15]. The authors observed that Urticaceae pollen grains could be distinguished with >98% accuracy. Moreover, the model could distinguish genera on before unseen Urticaceae pollen collected from aerobiological samples [15]. Olsson et al. [85] trained CNN models on 122,000 pollen grains, obtaining an accuracy of 98% for 83 species. Nevertheless, the accuracy dropped to 41% when individual reference samples from different flowers were kept separate [85]. Samonte et al. [86] developed a web-based application for food recommendation specialized in allergy information. In this application, restaurants would upload their menu and the individuals could make their choices based on potential known allergies. A selection of ML models’ outcomes in allergology is presented in Table 3.

ML learning frameworks have been developed specifically for allergy diagnoses, aiming to support junior clinicians and specialists in their decision-making tasks [94]. The main objective was to assist the management of complex cases, with multiple allergies, rather than focusing on easily diagnosable primary allergies. The framework includes a data cleaning module and utilizes modified sampling techniques in the data sampling module to improve the quality of intradermal test data. These processing steps significantly enhance the performance of the learning algorithms. Moreover, the adoption of a cross-validation approach ensures that the learning algorithms avoid overfitting the training data. Notably, ensemble classification approaches demonstrate a superior performance compared to traditional methods. The random forest classifier, employing constant strategy sampling, demonstrated superior sensitivity compared to all other cases [94].

To further improve the efficiency of the allergy diagnosis support system, meta-heuristic data-processing techniques can be employed. In addition to data cleaning and sampling, incorporating data transformation methods, such as feature selection, can be beneficial. Including prognosis details, treatment outcomes, and patient feedback will enhance the relevance of the system.

ML algorithms presented high accuracy and efficiency in identification of systemic lupus erythematosus (SLE) and neuropsychiatric systemic lupus erythematosus [95,96], as well as distinguishing patients with SLE and other major chronic autoimmune diseases, such as rheumatoid arthritis and multiple sclerosis, in the early stages [97]. Ali et al. [8] used a transcriptomic fragmentation model for biomarker detection in multiple sclerosis and rheumatoid arthritis, with a 96.45% accuracy. Li et al. [98] proposed combined proteomics and single-cell RNA sequencing to determine biomarker combinations for the diagnosis and activity monitoring in SLE patients; their model could efficiently assess disease exacerbation [98].

Martin-Gutierrez et al. [99] employed ML models to identify distinct immunologic signatures in subjects with primary Sjögren’s syndrome and SLE. The proposed model identified two distinct immune cell profiles, which could provide further directions in targeted therapy [99]. Therefore, AI can also be used to discover new treatments and predict drug efficiency for immune diseases by analyzing large amounts of genomic and proteomic data. ML algorithms predicted the efficiency of the etanercept in juvenile idiopathic arthritis using electronical medical records data, with a 75% sensitivity and 66.67% specificity [100].

Based on deep learning algorithms, Zeng et al. [101] developed deepDTnet, a model for target identification and drug repurposing, enclosing 15 types of cellular, phenotypic, genomic, and chemical profiles. Their proposed model showed a 96.3% accuracy in identifying novel molecular targets for known drugs [101]. Madhukar et al. [102] promoted BANDIT, a ML model that integrates multiple data types to identify connections between different drug types and classes and to predict drug binding targets.

AI has shown great potential in the field of vaccine development, as it can help to accelerate the identification of potential vaccine targets and the development of new vaccine candidates. Bukhari et al. [103] proposed a decision tree model for the prediction of novel and immunodominant Zika virus T-cell epitopes. The model showed a mean accuracy of 97.86%, with high possibilities in the development of Zika vaccines that target the predicted T-cell epitopes [103]. Arterolane and lucanthone were identified, based on a Bayesian ML model, as potential Ebola virus inhibitory agents [104].

AI-based models were also used for the COVID-19 vaccine development. Neural network-driven systems were used to discover T-cell epitopes for severe acute respiratory syndrome coronavirus 2 (SARS-CoV-2) [105]. The Long Short-Term Memory network was used to predict epitopes for Spike [106]. Pre-trained models were also used to predict molecular reactions in carbohydrate chemistry [107] and protein interaction [108].

Medical advances and high-tech developments, including AI, have led to significant advances in treating COVID-19. As a consequence of the inability to accurately and efficiently evaluate pulmonary lung CT data during the fight against COVID-19, Zhang et al. [109] developed a new system to analyze CT data of patients using deep learning and concluded that the right lower lobe of the lungs is the most common location for COVID-19 pneumonia. Additionally, Mohanty et al. [110] performed a quick intelligent screening for potential drugs to treat COVID-19 with a drug-repositioning method; this group was able to identify potential drugs using a combination of artificial intelligence and pharmacology, demonstrating the usefulness of this method to COVID-19 drug design and research. Moreover, other scholars have developed a platform based on AI learning and prediction models to identify the drugs on the market that may be useful for treating COVID-19; as a result, they found more than 80 drugs with considerable potential [111]. Stebbing et al. [112] analyzed existing anti-cytokine therapies, such as baricitinib, to explore new treatment options for COVID-19. Table 4 summarizes the developments of AI models in COVID-19 diagnoses, treatment, and prevention.

## 6. AI in Endocrinology

AI models have been analyzed in the diagnosis and treatment of multiple endocrinological conditions and pathologies, such as diabetes, thyroid disorders, reproductive impairments, or hormonal cancers.

One example of the use of AI in diabetes management is the development of closed-loop systems, also known as artificial pancreas systems. These systems use a combination of continuous glucose monitoring and insulin pumps to automatically regulate blood glucose levels. Neural network models had the most stable performance in such systems, being able to recover dynamics in short time intervals [127].

AI can also assist in the prediction of hypoglycemia, a common complication of diabetes. Continuous glucose monitoring data and clinical parameters are used in ML models to improve hypoglycemia prediction [128]. Ma et al. [129] introduced the MMTOP (multiple models for missing values at time of prediction) algorithm to predict the patient risk for severe hypoglycemia in the presence of incomplete data, with a cross-validated concordance index of 0.77 ± 0.03. Faruqui et al. [130] used deep learning algorithms to predict glucose levels in type 2 diabetes patients based on their diet, weight, glucose level from the day before, and physical activity.

Deep neural networks were able to predict gestational diabetes in early pregnancy, based on 73 variables, such as body mass index, 3,3,5′-triiodothyronine, or total thyroxin [131]. An unsupervised ML model was used to accurately classify four stable metabolic different obesity clusters: metabolic healthy obesity, hypermetabolic hyperuricemic obesity, hypermetabolic hyperinsulinemic obesity, and hypometabolic obesity [132]. Rein et al. [133] assessed the effect of a personalized postprandial-targeting diet (PPT) on glycemic control and metabolic health. The 6-month PPT intervention exerted significant improvement on glycated hemoglobin, fasting glucose, and triglycerides [133].

CNNs were used in thyroid pathology diagnoses. Yang et al. [134] proposed a deep learning framework trained on 508 ultrasound images to diagnose thyroid nodules. Their model showed an average accuracy of 98.4%. Islam et al. [135] compared 11 ML algorithms for thyroid risk prediction; the neural network classifier generated the highest accuracy over other ML techniques.

Reproductive health is a critical aspect of overall health and well-being, affecting individuals of all ages and genders. Hormonal imbalances and reproductive disorders can lead to infertility, pregnancy complications, and other health issues. In recent years, AI has emerged as a powerful tool for providing insights that may not be visible to human analyses. Polycystic ovary syndrome (PCOS) is a common hormonal disorder that affects up to 10% of women of a reproductive age [136]. It is characterized by irregular menstrual cycles, high levels of male hormones, and ovarian cysts [137]. The diagnosis of PCOS is currently based on clinical symptoms and a laboratory test, which can be subjective and lead to a misdiagnosis [138]. ML algorithms can assist in the diagnosis and management of PCOS. Suha and Islam [139] trained a CNN model on 594 ovary ultrasound images for cyst detection and PCOS diagnoses, with an accuracy of 99.89%. Zigarelli et al. [140] developed a self-diagnostic prediction model for PCOS, based on different variables, such as hirsutism, acne, an irregular menstrual cycle, the length of the menstrual cycle, and weight gain. Their model predicted a correct diagnosis with an accuracy ranging from 81% to 82.5%. Even if such self-diagnosis models can be useful in particular cases, which may include a lack of access to medical care or pandemic confinement, we consider that they should be taken “with a grain of salt”, as they cannot replace a professional diagnosis.

AI can also assist in the diagnosis and management of infertility by providing personalized recommendations. Ding et al. [141] compared seven ML methods in order to assess the ovarian reserve. The most accurate evaluation was provided by a light gradient boosting machine (LightGBM), which exerted the highest accuracy in the quantification of the ovarian reserve, especially in the 20–35 years age group [141]. The basal body temperature and heart rate were used to train ML algorithms, in order to predict the fertile window (a 72.51% accuracy) and menses (75.90% accuracy) [142].

A deep CNN was trained using single timepoint images of embryos, with an accuracy of 90% in choosing the highest quality embryo for in vitro fertilization (IVF); the trained CNN was also capable of predicting the potential of embryo implantation [143]. Louis et al. [144] analyzed different ML models (decision tree, random forest, and gradient booster) for IVF embryo selection. Their result revealed a lower peak accuracy of 65% [144].

## 7. AI in Dentistry

In recent years, AI has gained significant attention in the field of dentistry, with many researchers exploring its potential applications in diagnoses, treatment planning, and dental imaging analyses.

One of the most promising applications of AI in dentistry is in the area of dental imaging analyses; the use of AI algorithms has the potential to improve the accuracy and speed of an image analysis, with the identification of early signs of carious lesions, periapical lesions, or periodontal destruction. Furthermore, AI models can be trained to detect subtle changes in images over time, which may be indicative of the disease progression. Ameli et al. [145] used ordinal logistic regression and artificial neural networks to determine predictive relationships between the extracted patient chart data topics and oral health-related contributors; the authors observed that the risk for carious lesions, occlusal risk, biomechanical risk, gingival recession, periodontitis, and gingivitis were highly predictable using the extracted radiographic and treatment planning topics and chart information [145].

Carious lesions are usually detected by a clinical examination and X-ray visual analysis, highly depending on the experience of the specialists. Numerous studies focused on the AI models’ role in the early detection of carious lesions on dental X-rays (Table 5). Kühnisch et al. [146] proposed a CNN algorithm for carious lesion diagnoses on intraoral X-rays. Another study [147] compared the cost-effectiveness of AI for the detection of proximal caries with the diagnosis of dentists; the authors observed that the AI system was more effective and less expensive. Furthermore, AI algorithms can detect and analyze subtle changes in the periapical area, root canal anatomy, and bone structure [148].

A systematic review conducted by Mohammad-Rahimi et al. in 2022 assessed the DL capacity in various studies to detect carious lesions [149]. The authors observed different accuracies, mainly depending on the type of dataset, but with relatively high values: 71% to 96% on intra-oral photographs, 82% to 99.2% on periapical radiographs, 87.6% to 95.4% on bitewing radiographs, 68.0% to 78.0% on near-infrared transillumination images, 88.7% to 95.2% on optical coherence tomography images, and 86.1% to 96.1% on panoramic radiographs.

**Table 5:** AI outcomes in restorative dentistry.

Target	AI Model	Sample	Results	Study
Detection of simulated dental caries	Learning vector quantization	Teeth	AI is beneficial in diagnosing dental cavities.	Kositbowornchai et al., (2006) [150]
Dental caries detection	Adaptive dragonfly algorithm and neural network	120 dental images	Using the image processing method, a unique and upgraded model exhibits a much higher performance in detecting dental caries.	Patil et al., (2019) [151]
Dental caries detection	CNN	185 transillumination images	ROC of 83.6% for occlusal caries and ROC of 84.6% for proximal caries.	Casalegno et al., (2019) [152]
Root caries identification	ANN	357 variables in 5135 cases	97.1%, 95.1% precision, 99.6% sensitivity, 94.3% specificity.	Hung et al., (2019) [153]
To predict post-Streptococcus mutants	ANN	45 primary molars	Efficiency of 0.99033	Javed et al., (2020) [154]
Dental caries diagnosis	Backpropagation neural network	105 periapical X-rays	This model yielded an accuracy of 97.1%, a false positive (FP) rate of 2.8%, a receiver operating characteristic (ROC) area of 0.987, and a precision–recall curve (PRC) area of 0.987.	Geetha et al., (2020) [155]
Diagnosis of interproximal caries lesions	CNN	1000 digital bitewing radiographs	Total accuracy of 94.59% AUC of 7.19%	Bayraktar and Ayan (2022) [156]
Caries detection	CNN	2417 photographs	Accuracy of 92.5% (SE, 89.6; SP, 94.3; AUC, 0.964).	Kühnisch et al., (2022) [146]
Caries detection	CNN	226 extracted teeth 1319 teeth from 56 patients in vivo	Models trained and tested on in vivo data outperformed those trained and tested on in vitro data by a large margin. When evaluated in vitro, the models trained in vivo performed considerably lower (0.70 ± 0.01; *p* < 0.01). Similarly, when assessed in vivo, in vitro-taught models had considerably reduced accuracy (0.61 ± 0.04; *p* < 0.05).	Holtkamp et al., (2021) [157]
Detection and classification of dental restorations in panoramic radiography	Cubic support vector machine algorithm with error-correcting output codes	83 panoramic X-rays	Accuracy of 93.6%	Abdalla-Aslan et al., (2020) [158]

AI, artificial intelligence; ANN, artificial neural network; AUC, area under the curve; CBCT, cone-beam computed tomography; CI, confidence interval; CNN, convolutional neural network; ROC, receiver operating characteristic curve.

AI also found its way in periodontal diagnoses and prognoses [159,160,161]. AI models were used in order to detect the periodontal bone loss [159], periodontally compromised teeth [162], and even periodontal condition examination [163]. Troiano et al. [164] analyzed different AI models’ efficiency in assessing overall molar loss in patients after active periodontal treatment, with favorable results. A synthesis of the main outcomes in periodontics is presented in Table 6.

There is also great potential in AI for type recognition, success recognition, prediction, design, and optimization in dental implantology, as demonstrated by Revilla-Leon et al. [172]. AI systems can assist dentists and oral surgeons in planning the placement of dental implants by analyzing CBCT (cone-beam computed tomography) scans and identifying the optimal location, angulation, and size of implants, with a reduced risk of errors and complications [173]. AI can assist in the precise placement of dental implants during surgery by providing real-time guidance and feedback to clinicians (Table 7). AI can analyze CBCT scans and intraoperative data to help clinicians navigate the surgical site and ensure that the implants are placed in the optimal location and angulation [174].

AI can help dental professionals to design and create more accurate and personalized dental prosthetics for patients, by analyzing CBCT scans and digital impressions to create virtual 3D models. AI algorithms can also help to optimize the shape, size, and color of the restoration, ensuring a precise fit and a natural-looking appearance [179].

In orthodontics, AI systems have been applied in treatment planning and prediction of treatment outcomes, such as simulating changes in the appearance of pre- and post-treatment facial photographs [180] (Table 8). AI algorithms have been used in assessing the impact of orthodontic treatment, skeletal patterns, and anatomical landmarks in lateral cephalograms [181]. Other applications involved the diagnosis of the need for orthodontic treatment, tooth extraction determination in orthodontic treatments, or skeletal classification [182,183,184,185,186,187,188,189].

In oral and maxillofacial pathology, AI has been mainly researched for tumor and cancer detection based on radiographic, microscopic, and ultrasonographic images (Table 9). CNN models proved their accuracy end efficiency in detecting oral cancers [190]. Hung et al. [191] reviewed machine learning algorithms to predict oral cancer survival and factors affecting it; the authors concluded that cancer survival prediction and medical decision making were possible with the help of AI systems.

Neural network models have been investigated for their use in endodontic diagnoses and treatment planning (Table 10). Johari et al. (2017) used a probabilistic neural network (PNN) to diagnose vertical root fractures [198]. The model was trained on 240 radiographs (120 with intact dental roots and 120 with vertically fractured roots), as well as on cone-beam computed tomographies (CBCTs). The maximum accuracy, sensitivity, and specificity values in the three groups were 70.00, 97.78, and 67.7%, respectively, for radiographic images. When using CBCT images, the values were 96.6, 93.3, and 100%, respectively.

Therefore, various types of AI models are currently employed in the field of dentistry. Neural networks, including CNNs and ANNs, were among the earliest AI algorithms used. CNNs are primarily utilized for analyzing dental images. However, it is essential to implement more robust, reproducible, and standardized processes in the future, to ensure the usefulness, security, and widespread applicability of these models.

## 8. AI Advantages in Medicine

**Figure 3:**
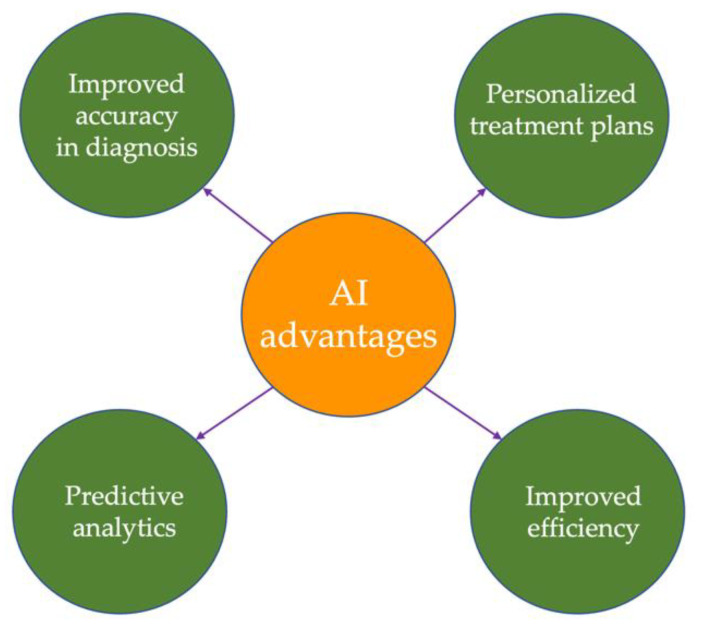
Advantages of AI in medicine.

Healthcare programs and procedures can benefit from the AI systems; the main advantages of using AI in medicine are presented in Figure 3. AI algorithms can process vast amounts of patient data and help physicians make more accurate and timely diagnoses. Therefore, they can reduce the risk of a misdiagnosis and improve patient outcomes [203], as well as reduce the initial process time up to 70% [204].

The 21st century belongs to personalized medicine, in which AI can play an important part. AI can help doctors tailor treatment plans to individual patients by analyzing patient data, medical records, and other relevant information.

AI algorithms can help healthcare providers identify patients who are at risk of developing complications or adverse reactions to treatment, allowing for early intervention and improved outcomes. Different AI models can automate many routine tasks, freeing up physicians and other healthcare professionals to focus on more complex cases and improving overall efficiency [205]. Continuous monitoring plays a crucial role in preventing potentially dangerous situations, allowing for the fine-tuning of ongoing treatments. This proactive approach enables a reduction of up to 40% in the total duration from the onset of illness to complete recovery. Moreover, AI facilitates the planning of more effective treatments while also accelerating the research and development of new medicines [206,207].

The ability to make early predictions holds tremendous potential in enhancing medical care, enabling healthcare providers to deliver more effective treatments and interventions. By leveraging intelligent phone-based prediction systems, patients gain the convenience of assessing their health condition without the need for in-person visits to the hospital. These systems use advanced algorithms and data analysis techniques to analyze symptoms, medical history, and other relevant factors, providing individuals with valuable insights into their current data [208].

Furthermore, AI can aid in identifying the root cause of various diseases. By analyzing a wide range of data, including genetic information, lifestyle factors, and environmental influences, these systems can uncover factors contributing to the development and progression of diseases [209].

Even though AI programs can be expensive, a global and perspective image of using AI mechanisms can generate cost savings in the end. This can be explained by the improved efficiency, a reduced risk of medical error, and a minimization of the need for expensive procedures and tests [210].

Moreover, AI systems can improve resource allocation. They can help healthcare providers identify areas where resources are needed most, such as high-risk patient populations or under-resourced communities [211].

Another potential advantage involves the accelerated drug discovery in which AI can be beneficial. AI algorithms are able to analyze large amounts of data to identify potential drug candidates and speed up the drug discovery process [32].

## 9. AI Disadvantages and Limitations in Medicine

**Figure 4:**
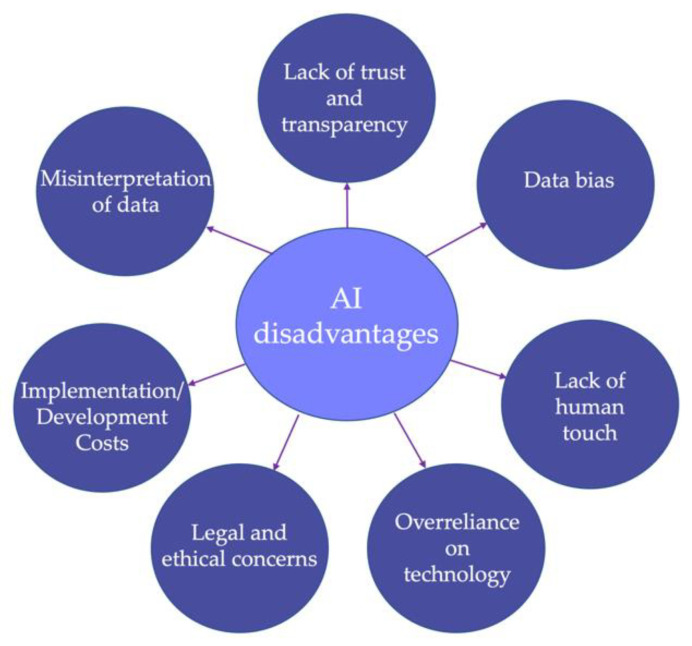
Disadvantages and limitations of AI in medicine.

Although AI has the potential to revolutionize healthcare and improve patient outcomes, there are also several disadvantages and limitations to its use in medicine (as synthesized in Figure 4). One of the major concerns with AI in healthcare is the lack of trust and transparency in the decision-making process [212]. Both healthcare providers and patients may be hesitant to rely on AI algorithms for a critical decision without a clear understanding of how the algorithm reached its conclusion. The level of trust that individuals have in AI is influenced by a diverse array of human characteristics. Factors such as education, user preferences, life experiences, and attitudes toward automation can all play a role in shaping trust [213]. People with a higher level of education or those who had positive experiences with AI technologies may be more inclined to trust AI systems.

Additionally, trust in AI is also influenced by the characteristics and attributes of the AI systems themselves. The degree of control that users have over the AI systems can impact trust levels. Users are more likely to trust AI systems that allow them to understand and influence the decision-making process. Transparent AI systems that provide clear explanations of their actions and reasoning can also enhance trust. On the other hand, highly complex AI systems that are difficult to comprehend may lower trust levels [214].

If users perceive AI systems to be prone to errors or potential harm, their trust may also be diminished. Ensuring the security and privacy of personal data handled by AI systems is essential for building trust.

Educating users about the capabilities and limitations of AI systems can favor trust levels. Developing systems with user-centric designs that prioritize transparency, explainability, and control can also foster trust. Additionally, addressing the ethical and regulatory concerns surrounding AI and implementing robust measures for data protection can enhance trust in AI technologies.

Another important limitation of AI in medicine is the need for large amounts of high-quality data to train AI algorithms [215]. The data must be carefully collected and curated to ensure that it is representative and unbiased. However, there may be challenges in collecting and sharing data across different healthcare systems due to issues of privacy, data ownership, and regulatory compliance. In addition, AI systems can be biased towards certain groups such as those with more available data [216]. This can lead to inaccuracies in diagnoses and treatment plans for underrepresented populations. Moreover, AI models can misinterpret data, leading to incorrect diagnoses or treatment plans. This is especially true when the data is noisy or incomplete, which is often the case in healthcare [5].

The development and implementation of AI systems can generate significant costs. This aspect can limit the access to these technologies, in particular in low-resource settings. The use of AI programs in healthcare has also raised several legal and ethical concerns, such as liability, privacy, and the potential for AI to replace human healthcare providers [217]. Another concern involves the overreliance on AI programs and models, which can lead to a reduction in critical thinking and clinical judgment among healthcare providers. This negative aspect affects both the professionals and the patient outcomes.

Ensuring robust data protection laws is of paramount importance in the era of big data, particularly in safeguarding the privacy of the patient. This situation raises concerns regarding the adequacy of existing regulations. It is imperative to address the shortcomings by encompassing health-related data that falls beyond the purview of current acts. Proactive measures are necessary to ensure comprehensive protection for health data, regardless of the entities involved in its collection, storage, and processing.

The sharing and regulation of disease-related data across multiple databases pose significant challenges due to the presence of personal information in patient records. This presents a complex landscape for software developers, who must navigate confidentiality regulations that can impede the development of AI. Privacy, in particular, is an important concern when dealing with health service data, as it represents the most private and personal information about individuals. Respecting confidentiality becomes an essential ethical principle in healthcare, intertwined with the autonomy, personal identity, and overall well-being of the patient [218].

On a different note, AI systems lack the empathy and personal touch that human healthcare providers can offer, which represent important aspects in patient care and satisfaction [13]. The role of human care providers extends beyond technical expertise. They engage in effective communication, and build trust with their patients. Instead of viewing the potential of intelligent artificial systems as replacements for human healthcare specialists, it is more appropriate to recognize the value of humans collaborating with these systems. The potential lies in integrating AI systems into healthcare workflows as tools to augment and enhance the capabilities of healthcare professionals.

Another three challenges of AI in healthcare include the black box problem, overfitting, and regulatory approval. A black box problem occurs when deep learning algorithms are unable to explain how their conclusions are reached. In the past, it was impossible to determine what imaging features were used in a process, how these were analyzed, and why the algorithm reached a particular conclusion [219]. Although the model could be simplified into a straightforward mathematical relationship linking symptoms to diagnoses, it is important to acknowledge that the underlying process may involve complex transformations that clinicians, and particularly patients, may struggle to comprehend. However, it is worth considering that the need for a complete understanding of the “black box” may not be necessary. In certain cases, positive results from randomized trials or other forms of testing could be sufficient evidence to demonstrate the safety and effectiveness of AI. While the internal workings of AI algorithms may remain complex and difficult to interpret, the focus can shift towards evaluating the overall performance and outcomes achieved through empirical validation.

Overfitting occurs when AI algorithms trained on one dataset have limited applicability to other datasets [220]. In this case, the algorithm has learned the statistical variations in the training data, rather than broad concepts required to solve a problem. The key determinant of overfitting is the overtraining of an algorithm on a specific dataset and several factors influence the likelihood of overfitting, including the size of the dataset, the extent of heterogeneity within the dataset, and the distribution of the data within the dataset.

Regulatory approval will pose a challenge for new AI algorithms. Medical AI, such as drugs and medical devices, require the FDA and regulation of other decisional organizations [221]. Due to the black box problem and overfitting, evaluators have difficulty understanding how algorithms work and whether their performance can be generalized to other datasets. AI tools are classified by the FDA based on three criteria: the risk to patient safety, predicate algorithms, and human input. In cases where algorithmic risks are high, such as diagnostic tools where a misdiagnosis would have severe consequences and where human input is minimal, premarket approval is conducted, which requires solid evidence that the tool is safe and effective from both non-clinical and clinical studies [6].

In addition, AI in medicine is still in the early stages of development, and there are limitations in terms of capabilities and accuracy of AI algorithms. While AI can analyze vast amounts of data quickly and efficiently, it may not be able to match the clinical expertise and intuition of human healthcare professionals. There may also be limitations in terms of the ability of AI to adapt to new situations and unexpected events, which can be critical in medical emergencies.

## 10. Discussion

### 10.1. Implications for Practice

The progress of science and technology has sparked a notable increase in the utilization of AI and other ML techniques in modern medical practice [222]. AI integration into healthcare has become an essential catalyst for advancements in medical diagnoses and healthcare innovation in the era of 4.0. With the aid of medical AI technologies, medical specialists now have access to algorithms and programs that enable them to analyze patients’ signs and symptoms, facilitating a deeper understanding of symbolic illness models and their interconnections.

Researchers in the field of AI have dedicated significant attention to diseases that are the leading causes of global mortality. It is projected that by 2030, chronic diseases will account for 80 percent of human lives lost worldwide, imposing substantial disease burdens on a global scale [223]. Consequently, researchers are leveraging cutting-edge technologies in the pursuit of early diagnoses and effective treatment approaches [224].

AI can assist a medical specialist by reducing the time spent on a diagnosis, allowing them to allocate more time to the patient’s treatment. Additionally, AI enables medical personnel to proactively identify potential medical errors by extracting precise data [205] (Lee & Yoon, 2021).

The active involvement of patients in the medical care process plays a vital role in ensuring a disease diagnosis and promoting an effective treatment. For instance, in the case of anticoagulant therapy for stroke patients, an AI platform increased treatment adherence by 50% [225].

### 10.2. Future Directions

There is a clear transformation taking place in the field of medicine as AI continues to make its mark, modernizing various traditional medical components. The constant advancement of AI in this domain ensures the ongoing development of algorithms that can provide accurate and reliable diagnoses without liability concerns. To enhance the quality standard of AI algorithms, input data need to be combined with pattern recognition that offers valuable insights into the future. Predictive diagnostics will play a role in authorizing insurance claims, shifting the focus towards illness prevention rather than solely treatment. Patients can expect a same-day diagnosis, authorization, and treatment facilitated by interconnected AI systems across clinics and insurers.

Furthermore, AI will contribute to the integration of treatment options across different healthcare areas. As data-driven therapy continues to rise, the boundaries between medical disciplines are gradually merging, leading to the integration of comprehensive healthcare services.

As AI continues to advance, there is potential to enhance the efficiency of processes throughout an extended public health continuum. This advancement could enable the implementation of personalized prediction and prevention approaches that can be tailored to individual needs and applied across different populations. Such an approach has the power to significantly expand the scope of public health, with the involvement of various organizations beyond traditional public health institutions.

The widespread implementation of AI in healthcare necessitates increased data sharing. However, certain stakeholders exhibit reluctance to share their data with other parties due to concerns regarding the security of sensitive personal or commercial information. Consequently, healthcare competition and antitrust laws must adapt to comprehend the nuances of big data and AI.

The ideal level of trust required between clinicians and AI systems for making accurate and reliable clinical decisions remains uncertain. Additionally, the connection between optimal trust in AI systems and their design attributes is yet to be determined. Addressing person-specific factors, such as significant variability associated with aleatory processes and the evolving capabilities of AI, is crucial in analyzing the problem.

### 10.3. Contribution to Literature and Limitations

The purpose of this research is to disseminate information and enhance overall awareness regarding the utilization of AI in the healthcare sector. The aim is to facilitate the implementation of prospective decision systems and enable an early prognosis for patients. Specifically, we sought to determine if there are broader issues associated with emerging technologies beyond healthcare service transformation. The following are key contributions of this review:

An overview and background of AI technology are provided to enhance the comprehension of cutting-edge concepts.

The context of AI in medical systems is explored, accompanied by a detailed discussion on ethical, legal, and trust-related concerns. This analysis aims to bolster public confidence in AI.

An examination of the reliability and utility of AI technology in healthcare applications is conducted.

After assessing the challenges and opportunities arising from the extensive integration of AI in healthcare, potential areas for future research are identified. These areas highlight avenues for further exploration and investigation.

Nevertheless, it is important to acknowledge the limitations of our research. Primarily, our study concentrated solely on a select few medical specialties within the vast realm of AI applications. This decision was driven by the authors’ intention to remain within the boundaries of their own field of expertise.

Additionally, it is worth noting that our research takes the form of a narrative review, primarily due to the heterogenous nature of the included studies. In order to provide quantifiable data, further investigations such as systematic reviews and meta-analyses are required to yield quantifiable data and enhance the level of evidence in this particular subject matter.

## 11. Conclusions

In this review, we have conducted an analysis of the applications and impacts of artificial intelligence and machine learning in healthcare infrastructure. We have explored the diverse uses of AI in the medical sector, including areas such as diagnoses, prognosis research, and development. The review highlights the significant contributions that AI systems have made in healthcare by enabling machines to emulate human-like behavior and exhibit intelligent capabilities.

This paper explores the benefits but also the challenges associated with integrating AI on a large scale in healthcare, and examines the ethical, legal, trust-building, and future implications of AI in the healthcare domain. Limitations of AI systems include the need for high-quality data, the potential for algorithmic bias, ethical concerns, and limitations in the capabilities and accuracy of AI algorithms. The insights presented in this paper aim to benefit the research community in developing AI systems tailored to healthcare, taking into account all the crucial aspects.

However, it is important to recognize the fact that our research focused solely on a limited number of medical specialties, a choice made to stay within the area of expertise of the authors. Furthermore, our research adopts a narrative format; additional investigations such as systematic reviews and meta-analyses are needed.

It is important to understand that we are still in the early stages of regulating the responsible design, development, and utilization of AI for healthcare, as the field is evolving rapidly. Nevertheless, it is our responsibility to conscientiously consider the ethical implications of implementing AI and to provide appropriate responses, even as the ethical landscape continues to evolve. As we continue to uncover their capabilities, AI systems have the potential to reshape healthcare delivery and improve patient outcomes.

## Figures and Tables

**Table 1:** Main AI systems used in medicine.

System	Abbreviation	Function
Artificial Neural Network	ANN	It is trained by processing examples, each of which contains a known “input” and “result,” forming probability-weighted associations between the two, which are stored within the data structure of the net itself [34].
Backpropagation Neural Network	-	Backpropagation is a process involved in training a neural network. It involves taking the error rate of a forward propagation and feeding this loss backward through the neural network layers to fine-tune the weights. Backpropagation is the essence of neural net training [35].
Bayesian Inference	-	It allows for an algorithm to make predictions based on prior beliefs. In Bayesian inference, the posterior distribution of predictors (derived from observed data) is updated based on new evidence [36].
Causal Associational Network	CASNET	This model consists of three main components: observations of a patient, pathophysiological states, and disease classifications. As observations are recorded, they are associated with the appropriate states [37].
Convolutional Neural Network	CNN	A network architecture for deep learning that learns directly from data. CNNs are particularly useful for finding patterns in images to recognize objects, classes, and categories. They can also be quite effective for classifying audio, time-series, and signal data [38].
Deep Neural Network	DNN	An ANN with multiple layers between the input and output layers. There are different types of neural networks but they always consist of the same components: neurons, synapses, weights, biases, and functions [39].
Light Gradient Boosting Machine	LightGBM	LightGBM is a gradient-boosting ensemble method that is based on decision trees. As with other decision tree-based methods, LightGBM can be used for both classification and regression. LightGBM is optimized for a high performance with distributed systems [40].
Multilayer Perceptron	MLP	A feedforward artificial neural network that generates a set of outputs from a set of inputs. An MLP is characterized by several layers of input nodes connected as a directed graph between the input and output layers. MLP uses backpropagation for training the network [41].
Natural Language Processing	NLP	It enables machines to understand the human language. Its goal is to build systems that can make sense of text and automatically perform tasks such as translation, a spell check, or topic classification [18].
Optimal Channel Networks	OCNet	Oriented spanning trees that reproduce all scaling features characteristic of real, natural river networks. As such, they can be used in a variety of numerical and laboratory experiments in the fields of hydrology, ecology, and epidemiology [42].
Probabilistic Neural Network	PNN	A feedforward neural network used to handle classification and pattern recognition problems [43].
Random Forest Models		An ensemble learning method for classification, regression, and other tasks that operates by constructing a multitude of decision trees at the training time [44].
Recurrent Neural Network	RNN	An ANN where connections between nodes can create a cycle, allowing output from some nodes to affect subsequent input to the same nodes. This allows it to exhibit temporal dynamic behavior [45].
Region-based Convolutional Neural Network	R-CNN	The key concept behind the R-CNN series is region proposals. Region proposals are used to localize objects within an image [46,47].
Support Vector Machine	SVM	A type of deep learning algorithm that performs supervised learning for classification or regression of data groups. In AI and machine learning, supervised learning systems provide both input and desired output data, which are labeled for classification [48].
Extreme Gradient Boosting	XGBoost	XGBoost, which stands for extreme gradient boosting, is a scalable, distributed gradient-boosted decision tree (GBDT) machine learning library. It provides parallel tree boosting and is the leading machine learning library for regression, classification, and ranking problems [49].

**Table 3:** Outcomes of ML models in allergology.

Target	Algorithm	Sample	Results	Study
Discriminating asthma from chronic obstructive pulmonary disease	Multinomial regression, gradient boosting, and recurrent neural networks	In total, 178,962 patients treated by two “R03” treatment prescriptions	The best models were obtained with the boosting approach and RNN, with an overall accuracy of 68%	Joumaa et al., (2022) [87]
Predicting pediatric asthma exacerbations	XGBoost (gradient-boosting decision trees)	Electronic health records for 5982 pediatric subjects	Sensitivity 70%, predictive values of 13.8% for 180-day outcomes and 2.9% for 30-day outcomes	Hurst et al., (2022) [88]
Diagnosis of AD on multiphoton tomography	CNN	In total, 3663 multiphoton tomography images from AD and healthy subjects	A correct diagnosis in 97.0% of all images Sensitivity of 0.966 Specificity of 0.977	Guimarães et al., (2020) [89]
Prediction of AD severity over time	Bayesian inference	Recordings of AD severity scores and treatments used by 59 and 334 pediatric subjects	Improvement of the chance-level forecast by 60%	Hurault et al., (2020) [90]
Phenotyping and identification of severity-associated factors in adolescent and adult patients with atopic dermatitis	ML-gradient boosting approach with cross-validation-based tuning multinomial logistic regression.	367 patients	The predictive performance of machine learning–gradient boosting vs. multinomial logistic regression differed only slightly (mean multiclass area under the curve value: 0.71 [95% CI, 0.69–0.72] vs. 0.68 [0.66–0.70], respectively	Maintz et al., (2021) [91]
Distinguishing different endotypes of CRSwNP based on clinical biomarkers	ANN logistic regression	In total, 15 clinical features from 60 healthy controls, 60 eCRSwNP, and 49 non-eCRSwNP	ANN models showed a better performance, significantly higher than those from the LR models (0.976 vs. 0.902, *p* = 0.048; 0.970 vs. 0.845, *p* = 0.011)	Zhou et al., (2023) [92]
Prediction of hypersensitivity to β-lactam	ANN logistic regression	Data records for 1271 allergic and non-allergic subjects	ANN: sensitivity of 89.5% and 81.1%, specificity of 86.1% and 97.9%, positive predictive values of 82.1% and 91.1% LR: sensitivity of 31.9% and specificity of 98.8%	Moreno et al., (2020) [93]

ANN, artificial neural network; AT, atopic dermatitis; CNN, convolutional neural network; CRSwNP, chronic rhinosinusitis with nasal polyps; eCRSwNP, eosinophilic CRSwNP; non-eCRSwNP, non-eosinophilic CRSwNP; ML, machine learning.

**Table 4:** AI outcomes in SARS-CoV-2.

AI Model	Results	Study
Diagnosis		
TopNetmAb model: Comprehensive topology-based AI.	Predict the binding free energy changes of S and ACE2/antibody complexes induced by mutations on the S RBD, of the Omicron variant.	Chen et al., (2022) [113]
DL method (3D-DL framework) for DNA sequence classification using CNN.	SARS-CoV-2 viral genomic sequencing. Viral evaluation accuracy > 99%.	Lopez-Rincon et al., (2021) [114]
Drug discovery		
DeepH-DTA: A squeezed-excited dense convolutional network for learning hidden representations within amino acid sequences.	Predict the affinity scores of drugs against SARS-CoV-2 amino acid sequences.	Abdel-Basset et al., (2020) [115]
Estimated drug–target interactions. A list of antiviral drugs was identified.	Molecule transformer–drug target interaction (MT-DTI).	Beck et al., (2020) [116]
AI-based generative network complex	Generate 15 potential drugs.	Gao et al., (2020) [117]
ChemAI; a deep neural network protocol on three drug discovery databases.	Generate 30,000 small compounds that are SARS-CoV-2 inhibitors.	Hofmarcher et al., (2020) [118]
ADQN-FBDD: An advanced deep Q-learning network with the fragment-based drug design (a model-free reinforcement learning algorithm).	Generate 47 lead compounds, targeting the SARS-CoV2 3C-like main protease.	Tang et al., (2020) [119]
Dense fully convolutional neural network (DFCNN). A list of chemical ligands and peptide drugs was provided.	Used four chemical compound and tripeptide databases to identify potential drugs for COVID-19.	Zhang et al., (2020) [109]
Generative DL. An AI-based drug discovery pipeline.	Generate inhibitors for the SARS-CoV-2 3CLpro.	Zhavoronkov et al., (2020) [120]
Vaccine development		
Bioinformatic tools and databases	Epitope vaccines were designed by using protein E as an antigenic site.	Abdelmageed et al., (2020) [121]
Computational methodology	Identify several epitopes in SARS-CoV-2 for the development of potential vaccines. S protein was identified as an immunogenic and effective vaccine candidate.	Fast et al., (2020) [122]
ML and reverse vaccinology	A cocktail vaccine with structural and non-structural proteins in which would accelerate efficient complementary immune responses.	Ong et al., (2020) [123]
Integrated bioinformatics pipeline that merges the prediction power of different software (in silico pipeline).	Predict the cross-reactivity of pre-existing vaccination interventions against SARS-CoV-2.	Russo et al., (2021) [124]
Immune informatics, reverse vaccinology, and molecular docking analysis.	Three epitope-based subunit vaccines were designated. Only one was reported as the best vaccine.	Sarkar et al., (2020) [125]
In silico approach. A molecular docking analysis.	A multi-epitopic vaccine candidate targeting the non-mutational immunogenic regions in envelope protein and surface glycoprotein of SARS-CoV-2.	Susithra Priyadarshni et al., (2021) [126]

3CLpro, 3C-like protease; AI, artificial intelligence; CNN, convolutional neural network; COVID-19, coronavirus disease 2019; DL, deep learning; HGAT, heterogeneous graph attention; ML, machine learning; RBD, receptor-binding domain; SARS-CoV-2, severe acute respiratory syndrome coronavirus 2.

**Table 6:** AI outcomes in periodontics.

Target	AI Model	Sample	Results	Study
To classify periodontitis by immune response profile to aggressive periodontitis or chronic periodontitis class.	MLP ANN	Data from 29 subjects	90–98% accuracy	Papantonopoulos et al., (2014) [165]
Diagnosis of periodontal diseases.	ANNs, decision trees, and support vector machine	Data from 150 patients	Performance was 98%. The poorest correlation between input and output variables was found in ANN, and its performance was assessed to be 46%.	Ozden et al., (2015) [166]
To identify and predict periodontally compromised teeth.	CNN encoder + three dense layers	1740 periapical X-rays	AUC of 73.4–82.6 [95% CI, 60.9–91.1] in predicting hopeless teeth.	J. H. Lee et al., (2018) [162]
To detect periodontal bone loss (PBL) on panoramic dental radiographs.	CNN + three dense layers	85 panoramic X-rays	Predictive accuracy was determined to be 81%, which is similar to the examiners.	Krois et al., (2019) [159]
Pre-emptive detection and diagnosis of periodontal disease and gingivitis by using intraoral images.	Faster R-CNN	134 photographs	Tooth detection accuracy of 100% to determine region of interest and 77.12% accuracy to detect inflammation.	Alalharith et al., (2020) [167]
Predicting periodontitis stage.	CNN	340 periapical X-rays	Accuracy of 68.3%	Danks et al., (2021) [168]
Predicting immunosuppression genes in periodontitis.	DisGeNet, HisgAtlas	Saliva	Accuracy of 92.78%	Ning et al., (2021) [169]
Clinical, immune, and microbial profiling of peri-implantitis patients against health.	CNN FARDEEP	Metabolites	Successfully used in logistic regression of plaque samples.	Wang et al., (2021) [170]
Research trialing different methods of segmentation to assess plaque on photographs of tooth surfaces (including ‘dye labelling’).	CNN OCNet, Anet	2884 photographs	AUC prediction of 87.11% for gingivitis and 80.11% for calculus.	Li et al., (2021) [171]

AI, artificial intelligence; ANN, artificial neural network; AUC, area under the curve; CBCT, cone-beam computed tomography; CI, confidence interval; CNN, convolutional neural network.

**Table 7:** AI outcomes in implantology.

Target	AI Model	Sample	Results	Study
Decision making in edentulous maxillary implant prostheses	ANN	Implant cases	Within a learning rate of 0.005, the network functioned admirably. The network’s accuracy for the new instances was 83.3%.	Sadighpour et al., (2014) [175]
To fabricate implant-supported monolithic zirconia crowns	ANN	Quality of the fabrication of the individual (zirconia abutment) and clinical parameters in subjects	AI appears to be a dependable solution for the restoration of single implants with zirconia crowns cemented on customized hybrid abutments using a fully digital process.	Lerner et al., (2020) [176]
Implant planning	CNN	75 CBCT images	There were statistically significant differences in bone thickness measurements between AI and manual measurements in all locations of the maxilla and mandible (*p* < 0.001). In addition, the proportion of correct recognition for canals was 72.2%, 66.4% for sinuses/fossae, and 95.3% for missing tooth areas.	Bayrakdar et al., (2021) [177]
Fractured dental implant detection and classification	CNN	Radiographic images of 251 intact and 194 fractured dental implants	When compared to fine-tuned and pre-trained VGGNet-19 and Google Net Inception-v3 architectures, automated DCNN architecture using periapical images demonstrated the highest and most reliable detection with an AUC of 0.984 [CI, 0.9–1.0] and classification performance AUC of 0.869 [CI, 0.778–0.929].	D. W. Lee et al., (2021) [178]

AI, artificial intelligence; ANN, artificial neural network; AUC, area under the curve; CBCT, cone-beam computed tomography; CI, confidence interval; CNN, convolutional neural network.

**Table 8:** AI outcomes in orthodontics.

Target	AI Model	Sample	Results	Study
To decide if extractions are necessary prior to orthodontic treatment	Backpropagation ANN	Data from 180 patients	Accuracy of 80% in predicting whether extraction or non-extraction treatments seemed appropriate for malocclusion patients aged 11 to 15 years.	Xie et al., (2010) [183]
To decide if extractions are necessary prior to orthodontic treatment	ANN	In total, 12 cephalometric variables and 6 indexes from 156 patients	Accuracy of 92%	Jung and Kim (2016) [184]
Determination of growth and development by cervical vertebrae stages	ANN	Cephalometric radiographs from 300 subjects	Mean accuracy of 77.2%	Kök et al., (2019) [187]
Osteoarthritis of the temporomandibular joint diagnosis	Logistic regression, random forest, LightGBM, XGBoost	CBCT blood serum saliva clinical investigation	Accuracy of 82.3%	Bianchi et al., (2020) [188]
Determination of growth and development periods	ANN	Cephalometric and hand–wrist radiographs in 419 subjects	Accuracy of 4.27%	Kök et al., (2021) [189]

AI, artificial intelligence; ANN, artificial neural network; CBCT, cone-beam computed tomography.

**Table 9:** AI outcomes in oral and maxillofacial surgery.

Target	AI Model	Sample	Results	Study
Lower-third-molar treatment-planning decisions	Neural networks	Data from 119 patients	Sensitivity of 0.78, which was slightly lower than the oral surgeon’s (0.88), but the difference was not significant, and a specificity of 0.98, which was lower than the oral surgeon’s (0.99) (*p* = NS).	Brickley and Shepherd (1996) [192]
To predict postoperative facial swelling following impacted mandibular third molar extraction	ANN	Data from 400 patients	This AI-based algorithm was 98% reliable in forecasting facial swelling after extraction of impacted third molar teeth.	Zhang et al., (2018) [193]
Ameloblastoma and keratocystic odontogenic tumor diagnosis	CNN	400/100 panoramic X-rays	The CNN had 81.8% sensitivity, 83.3% specificity, 83.0% accuracy, and a diagnostic time of 38 s, respectively.	Poedjiastoeti and Suebnukarn (2018) [194]
Evaluation of maxillary sinusitis on panoramic radiography	CNN	Panoramic X-rays from 400 maxillary sinusitis patients/400 healthy subjects	Accuracy of 87.5%, sensitivity of 86.7%, specificity of 88.3%, and area under the curve of 0.875 were obtained by the model.	Murata et al., (2019) [195]
Periapical disease detection	ANN	2902 panoramic X-rays	The deep learning method outperformed 14 of the 24 surgeons in the sample, with an average accuracy of 0.60 (0.04).	Endres et al., (2020) [196]
Automated detection of cyst and tumors of the jaw	CNN	1602 lesions on panoramic X-rays	Comparable with expert dentists.	Yang et al., (2020) [197]

AI, artificial intelligence; ANN, artificial neural network; CNN, convolutional neural network; NS, non-significant.

**Table 10:** AI outcomes in endodontics.

Target	AI Model	Sample	Results	Study
Locating the minor apical foramen	ANN	50 teeth	To enhance the accuracy of working length measurement using radiography, artificial neural networks can serve as a second opinion to find the apical foramen on radiographs.	Saghiri et al., (2012) [199]
Vertical root fracture detection	ANN	Digital X-rays: 50 sound and 150 vertical root fractures	Adequate sensitivity, specificity, and accuracy to be used as a model for vertical root fracture detection.	Kositbowornchai et al., (2013) [200]
Detecting vertical root fracture on X-ray images of endodontically treated and intact teeth	PNN	240 radiographs (120/120)	96.6% accuracy, 93.3% sensitivity, 100% specificity.	Johari et al., (2017) [198]
Detecting vertical root fracture on panoramic radiography	CNN	300 panoramic images	Precision of 0.93	Fukuda et al., (2020) [201]
To detect periapical pathosis	CNN	153 CBCT images	Accuracy of 92.8%	Orhan et al., (2020) [202]

AI, artificial intelligence; ANN, artificial neural network; CBCT, cone-beam computed tomography; CI, confidence interval; CNN, convolutional neural network; PNN, probabilistic neural network.

## Data Availability

Not applicable.

## Abbreviations

3CL^pro^	3C-like protease
AD	Atopic dermatitis
AF	Atrial fibrillation
AI	Artificial intelligence
ANN	Artificial neural network
AUC	Area under the curve
CASNET	Causal associational network
CBCT	Cone-beam computed tomography
ChatGBT	Chat generative pre-trained transformer
CHIEF	Congestive heart failure treatment performance measure information extraction framework
CI	Confidence interval
CNN	Convolutional neural network
COVID-19	Coronavirus disease 2019
CRSwNP	Chronic rhinosinusitis with nasal polyps
CT	Computed tomography
CVD	Cardiovascular disease
DL	Deep learning
DNN	Deep neural network
ECG	Electrocardiogram
eCRSwNP	Eosinophilic chronic rhinosinusitis with nasal polyps
EF	Ejection fraction
EMR	Electronic medical record
FFR	Fractional flow reserve
GCG	Gyrocardiography
HF	Heart failure
HGAT	Heterogeneous graph attention
IVF	In vitro fertilization
LightGBM	Light gradient boosting machine
LS	Longitudinal strain
LV	Left ventricular
ML	Machine learning
MLP	Multilayer perceptron
MMTOP	Multiple models for missing values at time of prediction
MRI	Magnetic resonance imaging
NLP	Natural language processing
OCNet	Optimal channel networks
PCA	Principal component analysis
PCOS	Polycystic ovary syndrome
PNN	Probabilistic neural network
PPG	Photoplethysmography
PPT	Postprandial targeting
RBD	Receptor-binding domain
R-CNN	Region-based convolutional neural network
RNA	Ribonucleic acid
ROC	Receiver operating characteristic
RV	Right ventricular
SARS-CoV-2	Severe acute respiratory syndrome coronavirus 2
SCG	Seismocardiography
SLE	Systemic lupus erythematosus
SNN	Simulated neural network
SVM	Support vector machine
TTE	Transthoracic echocardiography
VF	Ventricular fibrillation
VT	Ventricular tachycardia
XGBoost	Extreme gradient boosting

## References

[1] Zhang C., Lu Y. (2021). Study on Artificial Intelligence: The State of the Art and Future Prospects. J. Ind. Inf. Integr..

[2] Muftić F., Kadunić M., Mušinbegović A., Almisreb A.A. (2023). Exploring Medical Breakthroughs: A Systematic Review of ChatGPT Applications in Healthcare. Southeast Eur. J. Soft Comput..

[3] Esteva A., Chou K., Yeung S., Naik N., Madani A., Mottaghi A., Liu Y., Topol E., Dean J., Socher R. (2021). Deep Learning-Enabled Medical Computer Vision. npj Digit. Med..

[4] Zeng X., Wang F., Luo Y., Kang S., Tang J., Lightstone F.C., Fang E.F., Cornell W., Nussinov R., Cheng F. (2022). Deep Generative Molecular Design Reshapes Drug Discovery. Cell Rep. Med..

[5] Manickam P., Mariappan S.A., Murugesan S.M., Hansda S., Kaushik A., Shinde R., Thipperudraswamy S.P. (2022). Artificial Intelligence (AI) and Internet of Medical Things (IoMT) Assisted Biomedical Systems for Intelligent Healthcare. Biosensors.

[6] Kulkarni S., Seneviratne N., Baig M.S., Khan A.H.A. (2020). Artificial Intelligence in Medicine: Where Are We Now?. Acad. Radiol..

[7] Barragán-Montero A., Javaid U., Valdés G., Nguyen D., Desbordes P., Macq B., Willems S., Vandewinckele L., Holmström M., Löfman F. (2021). Artificial Intelligence and Machine Learning for Medical Imaging: A Technology Review. Phys. Med..

[8] Ali N.M., Shaheen M., Mabrouk M.S., Aborizka M. (2022). Machine Learning-Based Models for Detection of Biomarkers of Autoimmune Diseases by Fragmentation and Analysis of MiRNA Sequences. Appl. Sci..

[9] Kang J., Hanif M., Mirza E., Khan M.A., Malik M. (2021). Machine Learning in Primary Care: Potential to Improve Public Health. J. Med. Eng. Technol..

[10] Khan A.A., Laghari A.A., Awan S.A. (2021). Machine Learning in Computer Vision: A Review. EAI Endorsed Trans. Scalable Inf. Syst..

[11] Murugesan G., Ahmed T.I., Bhola J., Shabaz M., Singla J., Rakhra M., More S., Samori I.A. (2022). Fuzzy Logic-Based Systems for the Diagnosis of Chronic Kidney Disease. BioMed. Res. Int..

[12] Vlamou E., Papadopoulos B. (2019). Fuzzy Logic Systems and Medical Applications. AIMS Neurosci..

[13] Saibene A., Assale M., Giltri M. (2021). Expert Systems: Definitions, Advantages and Issues in Medical Field Applications. Expert Syst. Appl..

[14] Vrontis D., Christofi M., Pereira V., Tarba S., Makrides A., Trichina E. (2022). Artificial Intelligence, Robotics, Advanced Technologies and Human Resource Management: A Systematic Review. Int. J. Hum. Resour. Manag..

[15] Polling M., Li C., Cao L., Verbeek F., de Weger L.A., Belmonte J., De Linares C., Willemse J., de Boer H., Gravendeel B. (2021). Neural Networks for Increased Accuracy of Allergenic Pollen Monitoring. Sci. Rep..

[16] Ghorbani A., Ouyang D., Abid A., He B., Chen J.H., Harrington R.A., Liang D.H., Ashley E.A., Zou J.Y. (2020). Deep Learning Interpretation of Echocardiograms. npj Digit. Med..

[17] Kang Y., Cai Z., Tan C.-W., Huang Q., Liu H. (2020). Natural Language Processing (NLP) in Management Research: A Literature Review. J. Manag. Anal..

[18] Khurana D., Koli A., Khatter K., Singh S. (2023). Natural Language Processing: State of the Art, Current Trends and Challenges. Multimed. Tools Appl..

[19] Moran M.E. (2007). Evolution of Robotic Arms. J. Robot. Surg..

[20] Kaul V., Enslin S., Gross S.A. (2020). History of Artificial Intelligence in Medicine. Gastrointest. Endosc..

[21] Kuipers B., Feigenbaum E.A., Hart P.E., Nilsson N.J. (2017). Shakey: From Conception to History. AIMag.

[22] Kulikowski C.A. (2019). Beginnings of Artificial Intelligence in Medicine (AIM): Computational Artifice Assisting Scientific Inquiry and Clinical Art—With Reflections on Present AIM Challenges. Yearb. Med. Inf..

[23] Yang Y.J., Bang C.S. (2019). Application of Artificial Intelligence in Gastroenterology. World J. Gastroenterol..

[24] Ferrucci D., Levas A., Bagchi S., Gondek D., Mueller E.T. (2013). Watson: Beyond Jeopardy!. Artif. Intell..

[25] Bakkar N., Kovalik T., Lorenzini I., Spangler S., Lacoste A., Sponaugle K., Ferrante P., Argentinis E., Sattler R., Bowser R. (2018). Artificial Intelligence in Neurodegenerative Disease Research: Use of IBM Watson to Identify Additional RNA-Binding Proteins Altered in Amyotrophic Lateral Sclerosis. Acta Neuropathol..

[26] Comendador B.E.V., Francisco B.M.B., Medenilla J.S., Nacion S.M.T., Serac T.B.E. (2015). Pharmabot: A Pediatric Generic Medicine Consultant Chatbot. J. Autom. Control. Eng..

[27] Ni L., Lu C., Liu N., Liu J., Chen J., Theeramunkong T., Supnithi T., Tang X. (2017). MANDY: Towards a Smart Primary Care Chatbot Application. Knowledge and Systems Sciences, Proceedings of the Knowledge and Systems Sciences, Beijing, China, 11–12 June 2022.

[28] Rider N.L., Srinivasan R., Khoury P. (2020). Artificial Intelligence and the Hunt for Immunological Disorders. Curr. Opin. Allergy Clin. Immunol..

[29] Drummond D. (2021). Between Competence and Warmth: The Remaining Place of the Physician in the Era of Artificial Intelligence. npj Digit. Med..

[30] Gomes de Melo e Castro e Melo J.A., Faria Araújo N.M. (2020). Impact of the Fourth Industrial Revolution on the Health Sector: A Qualitative Study. Healthc. Inf. Res..

[31] Gonem S., Janssens W., Das N., Topalovic M. (2020). Applications of Artificial Intelligence and Machine Learning in Respiratory Medicine. Thorax.

[32] Nayarisseri A., Khandelwal R., Tanwar P., Madhavi M., Sharma D., Thakur G., Speck-Planche A., Singh S.K. (2021). Artificial Intelligence, Big Data and Machine Learning Approaches in Precision Medicine & Drug Discovery. Curr. Drug Targets.

[33] Saravi B., Hassel F., Ülkümen S., Zink A., Shavlokhova V., Couillard-Despres S., Boeker M., Obid P., Lang G.M. (2022). Artificial Intelligence-Driven Prediction Modeling and Decision Making in Spine Surgery Using Hybrid Machine Learning Models. J. Pers. Med..

[34] Si T., Bagchi J., Miranda P.B.C. (2022). Artificial Neural Network Training Using Metaheuristics for Medical Data Classification: An Experimental Study. Expert Syst. Appl..

[35] Ramya S.P., Sumitha B., Ranjani R., Ahamed M.A. A Comparative Study on Aspects Level Drug Reviews Using Back Propagation Neural Networks. Proceedings of the 2022 3rd International Conference on Electronics and Sustainable Communication Systems (ICESC).

[36] Van de Schoot R., Depaoli S., King R., Kramer B., Märtens K., Tadesse M.G., Vannucci M., Gelman A., Veen D., Willemsen J. (2021). Bayesian Statistics and Modelling. Nat. Rev. Methods Prim..

[37] Raita Y., Camargo C.A., Liang L., Hasegawa K. (2021). Big Data, Data Science, and Causal Inference: A Primer for Clinicians. Front. Med..

[38] Sarvamangala D.R., Kulkarni R.V. (2022). Convolutional Neural Networks in Medical Image Understanding: A Survey. Evol. Intell..

[39] Malhotra P., Gupta S., Koundal D., Zaguia A., Enbeyle W. (2022). Deep Neural Networks for Medical Image Segmentation. J. Healthc. Engin..

[40] Prediction Model of Hypertension Complications Based on GBDT and LightGBM—IOPscience. https://iopscience.iop.org/article/10.1088/1742-6596/1813/1/012008/meta.

[41] Li X.-D., Wang J.-S., Hao W.-K., Wang M., Zhang M. (2022). Multi-Layer Perceptron Classification Method of Medical Data Based on Biogeography-Based Optimization Algorithm with Probability Distributions. Appl. Soft. Comput..

[42] Yuan Y., Huang L., Guo J., Zhang C., Chen X., Wang J. (2018). OCNet: Object Context Network for Scene Parsing 2021. arXiv.

[43] Masegosa A.R., Cabañas R., Langseth H., Nielsen T.D., Salmerón A. (2021). Probabilistic Models with Deep Neural Networks. Entropy.

[44] MacEachern S.J., Forkert N.D. (2021). Machine Learning for Precision Medicine. Genome.

[45] Weerakody P.B., Wong K.W., Wang G., Ela W. (2021). A Review of Irregular Time Series Data Handling with Gated Recurrent Neural Networks. Neurocomputing.

[46] Choi B.W., Kang S., Kim H.W., Kwon O.D., Vu H.D., Youn S.W. (2021). Faster Region-Based Convolutional Neural Network in the Classification of Different Parkinsonism Patterns of the Striatum on Maximum Intensity Projection Images of [18F]FP-CIT Positron Emission Tomography. Diagnostics.

[47] Cha J.-Y., Yoon H.-I., Yeo I.-S., Huh K.-H., Han J.-S. (2021). Peri-Implant Bone Loss Measurement Using a Region-Based Convolutional Neural Network on Dental Periapical Radiographs. J. Clin. Med..

[48] K Faieq A., Mijwil M. (2022). Prediction of of Heart Diseases Utilising Support Vector Machine and Artificial Neural Network. Indones. J. Electr. Eng. Comput. Sci..

[49] Moore A., Bell M. (2022). XGBoost, A Novel Explainable AI Technique, in the Prediction of Myocardial Infarction: A UK Biobank Cohort Study. Clin. Med. Insights Cardiol..

[50] Attia Z.I., Kapa S., Lopez-Jimenez F., McKie P.M., Ladewig D.J., Satam G., Pellikka P.A., Enriquez-Sarano M., Noseworthy P.A., Munger T.M. (2019). Screening for Cardiac Contractile Dysfunction Using an Artificial Intelligence-Enabled Electrocardiogram. Nat. Med..

[51] Lippi G., Sanchis-Gomar F., Cervellin G. (2021). Global Epidemiology of Atrial Fibrillation: An Increasing Epidemic and Public Health Challenge. Int. J. Stroke.

[52] Wegner F.K., Plagwitz L., Doldi F., Ellermann C., Willy K., Wolfes J., Sandmann S., Varghese J., Eckardt L. (2022). Machine Learning in the Detection and Management of Atrial Fibrillation. Clin. Res. Cardiol..

[53] Zhang X., Gu K., Miao S., Zhang X., Yin Y., Wan C., Yu Y., Hu J., Wang Z., Shan T. (2020). Automated Detection of Cardiovascular Disease by Electrocardiogram Signal Analysis: A Deep Learning System. Cardiovasc. Diagn..

[54] AI Predicts Heart Disease Risk Using Single X-ray. https://press.rsna.org/timssnet/media/pressreleases/14_pr_target.cfm?id=2388.

[55] Hamatani Y., Nishi H., Iguchi M., Esato M., Tsuji H., Wada H., Hasegawa K., Ogawa H., Abe M., Fukuda S. (2022). Machine Learning Risk Prediction for Incident Heart Failure in Patients With Atrial Fibrillation. JACC Asia.

[56] Kee O.T., Harun H., Mustafa N., Abdul Murad N.A., Chin S.F., Jaafar R., Abdullah N. (2023). Cardiovascular Complications in a Diabetes Prediction Model Using Machine Learning: A Systematic Review. Cardiovasc. Diabetol..

[57] Nguyen M.T., Nguyen B.V., Kim K. (2018). Deep Feature Learning for Sudden Cardiac Arrest Detection in Automated External Defibrillators. Sci. Rep..

[58] Tison G.H., Sanchez J.M., Ballinger B., Singh A., Olgin J.E., Pletcher M.J., Vittinghoff E., Lee E.S., Fan S.M., Gladstone R.A. (2018). Passive Detection of Atrial Fibrillation Using a Commercially Available Smartwatch. JAMA Cardiol..

[59] Yang C., Aranoff N.D., Green P., Tavassolian N. A Binary Classification of Cardiovascular Abnormality Using Time-Frequency Features of Cardio-Mechanical Signals. Proceedings of the 2018 40th Annual International Conference of the IEEE Engineering in Medicine and Biology Society (EMBC).

[60] Wasserlauf J., You C., Patel R., Valys A., Albert D., Passman R. (2019). Smartwatch Performance for the Detection and Quantification of Atrial Fibrillation. Circ. Arrhythm. Electrophysiol..

[61] Kusunose K., Abe T., Haga A., Fukuda D., Yamada H., Harada M., Sata M. (2020). A Deep Learning Approach for Assessment of Regional Wall Motion Abnormality from Echocardiographic Images. JACC Cardiovasc. Imaging.

[62] Knackstedt C., Bekkers S.C.A.M., Schummers G., Schreckenberg M., Muraru D., Badano L.P., Franke A., Bavishi C., Omar A.M.S., Sengupta P.P. (2015). Fully Automated Versus Standard Tracking of Left Ventricular Ejection Fraction and Longitudinal Strain: The FAST-EFs Multicenter Study. J. Am. Coll. Cardiol..

[63] Itu L., Rapaka S., Passerini T., Georgescu B., Schwemmer C., Schoebinger M., Flohr T., Sharma P., Comaniciu D. (2016). A Machine-Learning Approach for Computation of Fractional Flow Reserve from Coronary Computed Tomography. J. Appl. Physiol..

[64] Zhang J., Gajjala S., Agrawal P., Tison G.H., Hallock L.A., Beussink-Nelson L., Lassen M.H., Fan E., Aras M.A., Jordan C. (2018). Fully Automated Echocardiogram Interpretation in Clinical Practice. Circulation.

[65] Retson T.A., Masutani E.M., Golden D., Hsiao A. (2020). Clinical Performance and Role of Expert Supervision of Deep Learning for Cardiac Ventricular Volumetry: A Validation Study. Radiol. Artif. Intell..

[66] Alzubaidi L., Zhang J., Humaidi A.J., Al-Dujaili A., Duan Y., Al-Shamma O., Santamaría J., Fadhel M.A., Al-Amidie M., Farhan L. (2021). Review of Deep Learning: Concepts, CNN Architectures, Challenges, Applications, Future Directions. J. Big Data.

[67] Wang Y., Luo J., Hao S., Xu H., Shin A.Y., Jin B., Liu R., Deng X., Wang L., Zheng L. (2015). NLP Based Congestive Heart Failure Case Finding: A Prospective Analysis on Statewide Electronic Medical Records. Int. J. Med. Inf..

[68] Meystre S.M., Kim Y., Gobbel G.T., Matheny M.E., Redd A., Bray B.E., Garvin J.H. (2017). Congestive Heart Failure Information Extraction Framework for Automated Treatment Performance Measures Assessment. J. Am. Med. Inf. Assoc..

[69] Ye C., Fu T., Hao S., Zhang Y., Wang O., Jin B., Xia M., Liu M., Zhou X., Wu Q. (2018). Prediction of Incident Hypertension Within the Next Year: Prospective Study Using Statewide Electronic Health Records and Machine Learning. J. Med. Internet Res..

[70] Samad M.D., Ulloa A., Wehner G.J., Jing L., Hartzel D., Good C.W., Williams B.A., Haggerty C.M., Fornwalt B.K. (2019). Predicting Survival From Large Echocardiography and Electronic Health Record Datasets: Optimization With Machine Learning. JACC Cardiovasc. Imaging.

[71] Kwon J.-M., Kim K.-H., Jeon K.-H., Park J. (2019). Deep Learning for Predicting In-Hospital Mortality among Heart Disease Patients Based on Echocardiography. Echocardiography.

[72] Lu J., Hutchens R., Hung J., Bennamoun M., McQuillan B., Briffa T., Sohel F., Murray K., Stewart J., Chow B. (2022). Performance of Multilabel Machine Learning Models and Risk Stratification Schemas for Predicting Stroke and Bleeding Risk in Patients with Non-Valvular Atrial Fibrillation. Comput. Biol. Med..

[73] Steele A.J., Denaxas S.C., Shah A.D., Hemingway H., Luscombe N.M. (2018). Machine Learning Models in Electronic Health Records Can Outperform Conventional Survival Models for Predicting Patient Mortality in Coronary Artery Disease. PLoS ONE.

[74] Chi C.-L., Wang J., Ying Yew P., Lenskaia T., Loth M., Mani Pradhan P., Liang Y., Kurella P., Mehta R., Robinson J.G. (2022). Producing Personalized Statin Treatment Plans to Optimize Clinical Outcomes Using Big Data and Machine Learning. J. Biomed. Inf..

[75] Malizia V., Cilluffo G., Fasola S., Ferrante G., Landi M., Montalbano L., Licari A., La Grutta S. (2022). Endotyping Allergic Rhinitis in Children: A Machine Learning Approach. Pediatr. Allergy Immunol..

[76] Yang J., Zhang M., Liu P., Yu S. (2022). Multi-Label Rhinitis Prediction Using Ensemble Neural Network Chain with Pre-Training. Appl. Soft Comput..

[77] Bhardwaj P., Tyagi A., Tyagi S., Antão J., Deng Q. (2023). Machine Learning Model for Classification of Predominantly Allergic and Non-Allergic Asthma among Preschool Children with Asthma Hospitalization. J. Asthma.

[78] Van Breugel M., Qi C., Xu Z., Pedersen C.-E.T., Petoukhov I., Vonk J.M., Gehring U., Berg M., Bügel M., Carpaij O.A. (2022). Nasal DNA Methylation at Three CpG Sites Predicts Childhood Allergic Disease. Nat. Commun..

[79] Proper S.P., Azouz N.P., Mersha T.B. (2021). Achieving Precision Medicine in Allergic Disease: Progress and Challenges. Front. Immunol..

[80] Wu C., Lu P., Xu F., Duan J., Hua X., Shabaz M. (2021). The Prediction Models of Anaphylactic Disease. Inf. Med. Unlocked.

[81] Khoury P., Srinivasan R., Kakumanu S., Ochoa S., Keswani A., Sparks R., Rider N.L. (2022). A Framework for Augmented Intelligence in Allergy and Immunology Practice and Research-A Work Group Report of the AAAAI Health Informatics, Technology, and Education Committee. J. Allergy Clin. Immunol. Pract..

[82] Ridolo E., Incorvaia C., Heffler E., Cavaliere C., Paoletti G., Canonica G.W. (2022). The Present and Future of Allergen Immunotherapy in Personalized Medicine. J. Pers. Med..

[83] Wild C.P. (2005). Complementing the Genome with an “Exposome”: The Outstanding Challenge of Environmental Exposure Measurement in Molecular Epidemiology. Cancer Epidemiol. Biomark. Prev..

[84] Tunyasuvunakool K., Adler J., Wu Z., Green T., Zielinski M., Žídek A., Bridgland A., Cowie A., Meyer C., Laydon A. (2021). Highly Accurate Protein Structure Prediction for the Human Proteome. Nature.

[85] Olsson O., Karlsson M., Persson A.S., Smith H.G., Varadarajan V., Yourstone J., Stjernman M. (2021). Efficient, Automated and Robust Pollen Analysis Using Deep Learning. Methods Ecol. Evol..

[86] Samonte M.J., Sunga C.F., Samonte D. AlleRT: Food Recommender Web Application with Allergy Filtration. Proceedings of the 5th European International Conference on Industrial Engineering and Operations Management.

[87] Joumaa H., Sigogne R., Maravic M., Perray L., Bourdin A., Roche N. (2022). Artificial Intelligence to Differentiate Asthma from COPD in Medico-Administrative Databases. BMC Pulm. Med..

[88] Hurst J.H., Zhao C., Hostetler H.P., Ghiasi Gorveh M., Lang J.E., Goldstein B.A. (2022). Environmental and Clinical Data Utility in Pediatric Asthma Exacerbation Risk Prediction Models. BMC Med. Inf. Decis. Mak..

[89] Guimarães P., Batista A., Zieger M., Kaatz M., Koenig K. (2020). Artificial Intelligence in Multiphoton Tomography: Atopic Dermatitis Diagnosis. Sci. Rep..

[90] Hurault G., Domínguez-Hüttinger E., Langan S.M., Williams H.C., Tanaka R.J. (2020). Personalized Prediction of Daily Eczema Severity Scores Using a Mechanistic Machine Learning Model. Clin. Exp. Allergy.

[91] Maintz L., Welchowski T., Herrmann N., Brauer J., Kläschen A.S., Fimmers R., Schmid M., Bieber T., Schmid-Grendelmeier P., CK-CARE Study Group (2021). Machine Learning-Based Deep Phenotyping of Atopic Dermatitis: Severity-Associated Factors in Adolescent and Adult Patients. JAMA Derm..

[92] Zhou H., Fan W., Qin D., Liu P., Gao Z., Lv H., Zhang W., Xiang R., Xu Y. (2023). Development, Validation and Comparison of Artificial Neural Network and Logistic Regression Models Predicting Eosinophilic Chronic Rhinosinusitis with Nasal Polyps. Allergy Asthma Immunol. Res..

[93] Moreno E.M., Moreno V., Laffond E., Gracia-Bara M.T., Muñoz-Bellido F.J., Macías E.M., Curto B., Campanon M.V., de Arriba S., Martin C. (2020). Usefulness of an Artificial Neural Network in the Prediction of β-Lactam Allergy. J. Allergy Clin. Immunol. Pract..

[94] Ramisetty K., Christopher J., Panda S., Lazarus Y. (2021). Machine Learning and XAI Approaches for Allergy Diagnosis. Biomed. Signal Process Control.

[95] Jorge A., Castro V.M., Barnado A., Gainer V., Hong C., Cai T., Cai T., Carroll R., Denny J.C., Crofford L. (2019). Identifying Lupus Patients in Electronic Health Records: Development and Validation of Machine Learning Algorithms and Application of Rule-Based Algorithms. Semin. Arthritis Rheum..

[96] Zhou Y., Wang M., Zhao S., Yan Y. (2022). Machine Learning for Diagnosis of Systemic Lupus Erythematosus: A Systematic Review and Meta-Analysis. Comput. Intell. Neurosci..

[97] Ma Y., Chen J., Wang T., Zhang L., Xu X., Qiu Y., Xiang A.P., Huang W. (2022). Accurate Machine Learning Model to Diagnose Chronic Autoimmune Diseases Utilizing Information From B Cells and Monocytes. Front. Immunol..

[98] Li Y., Ma C., Liao S., Qi S., Meng S., Cai W., Dai W., Cao R., Dong X., Krämer B.K. (2022). Combined Proteomics and Single Cell RNA-Sequencing Analysis to Identify Biomarkers of Disease Diagnosis and Disease Exacerbation for Systemic Lupus Erythematosus. Front. Immunol..

[99] Martin-Gutierrez L., Peng J., Thompson N.L., Robinson G.A., Naja M., Peckham H., Wu W., J’bari H., Ahwireng N., Waddington K.E. (2021). Stratification of Patients With Sjögren’s Syndrome and Patients With Systemic Lupus Erythematosus According to Two Shared Immune Cell Signatures, With Potential Therapeutic Implications. Arthritis Rheumatol..

[100] Mo X., Chen X., Ieong C., Zhang S., Li H., Li J., Lin G., Sun G., He F., He Y. (2020). Early Prediction of Clinical Response to Etanercept Treatment in Juvenile Idiopathic Arthritis Using Machine Learning. Front. Pharm..

[101] Zeng X., Zhu S., Lu W., Liu Z., Huang J., Zhou Y., Fang J., Huang Y., Guo H., Li L. (2020). Target Identification among Known Drugs by Deep Learning from Heterogeneous Networks. Chem. Sci..

[102] Madhukar N.S., Khade P.K., Huang L., Gayvert K., Galletti G., Stogniew M., Allen J.E., Giannakakou P., Elemento O. (2019). A Bayesian Machine Learning Approach for Drug Target Identification Using Diverse Data Types. Nat. Commun..

[103] Bukhari S.N.H., Webber J., Mehbodniya A. (2022). Decision Tree Based Ensemble Machine Learning Model for the Prediction of Zika Virus T-Cell Epitopes as Potential Vaccine Candidates. Sci. Rep..

[104] Anantpadma M., Lane T., Zorn K.M., Lingerfelt M.A., Clark A.M., Freundlich J.S., Davey R.A., Madrid P.B., Ekins S. (2019). Ebola Virus Bayesian Machine Learning Models Enable New in Vitro Leads. ACS Omega.

[105] Crooke S.N., Ovsyannikova I.G., Kennedy R.B., Poland G.A. (2020). Immunoinformatic Identification of B Cell and T Cell Epitopes in the SARS-CoV-2 Proteome. Sci. Rep..

[106] Abbasi B.A., Saraf D., Sharma T., Sinha R., Singh S., Sood S., Gupta P., Gupta A., Mishra K., Kumari P. (2022). Identification of Vaccine Targets & Design of Vaccine against SARS-CoV-2 Coronavirus Using Computational and Deep Learning-Based Approaches. PeerJ.

[107] Nambiar A., Liu S., Heflin M., Forsyth J.M., Maslov S., Hopkins M., Ritz A. (2023). Transformer Neural Networks for Protein Family and Interaction Prediction Tasks. J. Comput. Biol..

[108] Pesciullesi G., Schwaller P., Laino T., Reymond J.-L. (2020). Transfer Learning Enables the Molecular Transformer to Predict Regio- and Stereoselective Reactions on Carbohydrates. Nat. Commun..

[109] Zhang H.-T., Zhang J.-S., Zhang H.-H., Nan Y.-D., Zhao Y., Fu E.-Q., Xie Y.-H., Liu W., Li W.-P., Zhang H.-J. (2020). Automated Detection and Quantification of COVID-19 Pneumonia: CT Imaging Analysis by a Deep Learning-Based Software. Eur. J. Nucl. Med. Mol. Imaging.

[110] Mohanty S., Harun Ai Rashid M., Mridul M., Mohanty C., Swayamsiddha S. (2020). Application of Artificial Intelligence in COVID-19 Drug Repurposing. Diabetes Metab. Syndr..

[111] Liu P.-R., Lu L., Zhang J.-Y., Huo T.-T., Liu S.-X., Ye Z.-W. (2021). Application of Artificial Intelligence in Medicine: An Overview. Curr. Med. Sci..

[112] Stebbing J., Krishnan V., de Bono S., Ottaviani S., Casalini G., Richardson P.J., Monteil V., Lauschke V.M., Mirazimi A., Youhanna S. (2020). Mechanism of Baricitinib Supports Artificial Intelligence-Predicted Testing in COVID-19 Patients. EMBO Mol. Med..

[113] Chen J., Wang R., Gilby N.B., Wei G.-W. (2022). Omicron Variant (B.1.1.529): Infectivity, Vaccine Breakthrough, and Antibody Resistance. J. Chem. Inf. Model.

[114] Lopez-Rincon A., Tonda A., Mendoza-Maldonado L., Mulders D.G.J.C., Molenkamp R., Perez-Romero C.A., Claassen E., Garssen J., Kraneveld A.D. (2021). Classification and Specific Primer Design for Accurate Detection of SARS-CoV-2 Using Deep Learning. Sci. Rep..

[115] Abdel-Basset M., Hawash H., Elhoseny M., Chakrabortty R.K., Ryan M. (2020). DeepH-DTA: Deep Learning for Predicting Drug-Target Interactions: A Case Study of COVID-19 Drug Repurposing. IEEE Access.

[116] Beck B.R., Shin B., Choi Y., Park S., Kang K. (2020). Predicting Commercially Available Antiviral Drugs That May Act on the Novel Coronavirus (SARS-CoV-2) through a Drug-Target Interaction Deep Learning Model. Comput. Struct. Biotechnol. J..

[117] Gao K., Nguyen D.D., Wang R., Wei G.-W. (2020). Machine Intelligence Design of 2019-NCoV Drugs. bioRxiv.

[118] Hofmarcher M., Mayr A., Rumetshofer E., Ruch P., Renz P., Schimunek J., Seidl P., Vall A., Widrich M., Hochreiter S. (2020). Large-Scale Ligand-Based Virtual Screening for SARS-CoV-2 Inhibitors Using Deep Neural Networks. arXiv.

[119] Tang B., He F., Liu D., Fang M., Wu Z., Xu D. (2022). AI-Aided Design of Novel Targeted Covalent Inhibitors against SARS-CoV-2. Biomolecules.

[120] Zhavoronkov A., Aladinskiy V., Zhebrak A., Zagribelnyy B., Terentiev V., Bezrukov D.S., Polykovskiy D., Shayakhmetov R., Filimonov A., Orekhov P. (2020). Potential COVID-2019 3C-like Protease Inhibitors Designed Using Generative Deep Learning Approaches. ChemRxiv.

[121] Abdelmageed M.I., Abdelmoneim A.H., Mustafa M.I., Elfadol N.M., Murshed N.S., Shantier S.W., Makhawi A.M. (2020). Design of a Multiepitope-Based Peptide Vaccine against the E Protein of Human COVID-19: An Immunoinformatics Approach. Biomed. Res. Int..

[122] Fast E., Altman R.B., Chen B. (2020). Potential T-Cell and B-Cell Epitopes of 2019-NCoV. bioRxiv.

[123] Ong E., Wong M.U., Huffman A., He Y. (2020). COVID-19 Coronavirus Vaccine Design Using Reverse Vaccinology and Machine Learning. bioRxiv.

[124] Russo G., Di Salvatore V., Sgroi G., Parasiliti Palumbo G.A., Reche P.A., Pappalardo F. (2022). A Multi-Step and Multi-Scale Bioinformatic Protocol to Investigate Potential SARS-CoV-2 Vaccine Targets. Brief Bioinform..

[125] Sarkar B., Ullah M.A., Johora F.T., Taniya M.A., Araf Y. (2020). The Essential Facts of Wuhan Novel Coronavirus Outbreak in China and Epitope-Based Vaccine Designing against COVID-19. bioRxiv.

[126] Susithra Priyadarshni M., Isaac Kirubakaran S., Harish M.C. (2022). In Silico Approach to Design a Multi-Epitopic Vaccine Candidate Targeting the Non-Mutational Immunogenic Regions in Envelope Protein and Surface Glycoprotein of SARS-CoV-2. J. Biomol. Struct. Dyn..

[127] Zhang M., Flores K.B., Tran H.T. (2021). Deep Learning and Regression Approaches to Forecasting Blood Glucose Levels for Type 1 Diabetes. Biomed. Signal Process. Control.

[128] Mujahid O., Contreras I., Vehi J. (2021). Machine Learning Techniques for Hypoglycemia Prediction: Trends and Challenges. Sensors.

[129] Ma S., Schreiner P.J., Seaquist E.R., Ugurbil M., Zmora R., Chow L.S. (2020). Multiple Predictively Equivalent Risk Models for Handling Missing Data at Time of Prediction: With an Application in Severe Hypoglycemia Risk Prediction for Type 2 Diabetes. J. Biomed. Inf..

[130] Faruqui S.H.A., Du Y., Meka R., Alaeddini A., Li C., Shirinkam S., Wang J. (2019). Development of a Deep Learning Model for Dynamic Forecasting of Blood Glucose Level for Type 2 Diabetes Mellitus: Secondary Analysis of a Randomized Controlled Trial. JMIR Mhealth Uhealth.

[131] Wu Y.-T., Zhang C.-J., Mol B.W., Kawai A., Li C., Chen L., Wang Y., Sheng J.-Z., Fan J.-X., Shi Y. (2021). Early Prediction of Gestational Diabetes Mellitus in the Chinese Population via Advanced Machine Learning. J. Clin. Endocrinol. Metab..

[132] Lin Z., Feng W., Liu Y., Ma C., Arefan D., Zhou D., Cheng X., Yu J., Gao L., Du L. (2021). Machine Learning to Identify Metabolic Subtypes of Obesity: A Multi-Center Study. Front. Endocrinol..

[133] Rein M., Ben-Yacov O., Godneva A., Shilo S., Zmora N., Kolobkov D., Cohen-Dolev N., Wolf B.-C., Kosower N., Lotan-Pompan M. (2022). Effects of Personalized Diets by Prediction of Glycemic Responses on Glycemic Control and Metabolic Health in Newly Diagnosed T2DM: A Randomized Dietary Intervention Pilot Trial. BMC Med..

[134] Yang J., Shi X., Wang B., Qiu W., Tian G., Wang X., Wang P., Yang J. (2022). Ultrasound Image Classification of Thyroid Nodules Based on Deep Learning. Front. Oncol..

[135] Islam S.S., Haque M.S., Miah M.S.U., Sarwar T.B., Nugraha R. (2022). Application of Machine Learning Algorithms to Predict the Thyroid Disease Risk: An Experimental Comparative Study. PeerJ Comput. Sci..

[136] Ndefo U.A., Eaton A., Green M.R. (2013). Polycystic Ovary Syndrome: A Review of Treatment Options with a Focus on Pharmacological Approaches. P T.

[137] Azziz R. (2018). Polycystic Ovary Syndrome. Obs. Gynecol..

[138] Ni C.-M., Huang W.-L., Jiang Y.-M., Xu J., Duan R., Zhu Y.-L., Zhu X.-P., Fan X.-M., Luo G.-A., Wang Y.-M. (2020). Improving the Accuracy and Efficacy of Diagnosing Polycystic Ovary Syndrome by Integrating Metabolomics with Clinical Characteristics: Study Protocol for a Randomized Controlled Trial. Trials.

[139] Suha S.A., Islam M.N. (2022). An Extended Machine Learning Technique for Polycystic Ovary Syndrome Detection Using Ovary Ultrasound Image. Sci. Rep..

[140] Zigarelli A., Jia Z., Lee H. (2022). Machine-Aided Self-Diagnostic Prediction Models for Polycystic Ovary Syndrome: Observational Study. JMIR Res..

[141] Ding T., Ren W., Wang T., Han Y., Ma W., Wang M., Fu F., Li Y., Wang S. (2023). Assessment and Quantification of Ovarian Reserve on the Basis of Machine Learning Models. Front. Endocrinol..

[142] Yu J.-L., Su Y.-F., Zhang C., Jin L., Lin X.-H., Chen L.-T., Huang H.-F., Wu Y.-T. (2022). Tracking of Menstrual Cycles and Prediction of the Fertile Window via Measurements of Basal Body Temperature and Heart Rate as Well as Machine-Learning Algorithms. Reprod. Biol. Endocrinol..

[143] Bormann C.L., Kanakasabapathy M.K., Thirumalaraju P., Gupta R., Pooniwala R., Kandula H., Hariton E., Souter I., Dimitriadis I., Ramirez L.B. (2020). Performance of a Deep Learning Based Neural Network in the Selection of Human Blastocysts for Implantation. eLife.

[144] Louis C.M., Handayani N., Aprilliana T., Polim A.A., Boediono A., Sini I. (2023). Genetic Algorithm-Assisted Machine Learning for Clinical Pregnancy Prediction in in Vitro Fertilization. AJOG Glob. Rep..

[145] Ameli N., Gibson M.P., Khanna A., Howey M., Lai H. (2022). An Application of Machine Learning Techniques to Analyze Patient Information to Improve Oral Health Outcomes. Front. Dent. Med..

[146] Kühnisch J., Meyer O., Hesenius M., Hickel R., Gruhn V. (2022). Caries Detection on Intraoral Images Using Artificial Intelligence. J. Dent. Res..

[147] Schwendicke F., Rossi J.G., Göstemeyer G., Elhennawy K., Cantu A.G., Gaudin R., Chaurasia A., Gehrung S., Krois J. (2021). Cost-Effectiveness of Artificial Intelligence for Proximal Caries Detection. J. Dent. Res..

[148] Agrawal P., Nikhade P. (2022). Artificial Intelligence in Dentistry: Past, Present, and Future. Cureus.

[149] Mohammad-Rahimi H., Motamedian S.R., Rohban M.H., Krois J., Uribe S.E., Mahmoudinia E., Rokhshad R., Nadimi M., Schwendicke F. (2022). Deep Learning for Caries Detection: A Systematic Review. J. Dent..

[150] Kositbowornchai S., Siriteptawee S., Plermkamon S., Bureerat S., Chetchotsak D. (2006). An Artificial Neural Network for Detection of Simulated Dental Caries. Int. J. Comput. Assist. Radiol. Surg..

[151] Patil S., Kulkarni V., Bhise A. (2019). Algorithmic Analysis for Dental Caries Detection Using an Adaptive Neural Network Architecture. Heliyon.

[152] Casalegno F., Newton T., Daher R., Abdelaziz M., Lodi-Rizzini A., Schürmann F., Krejci I., Markram H. (2019). Caries Detection with Near-Infrared Transillumination Using Deep Learning. J. Dent. Res..

[153] Hung M., Voss M.W., Rosales M.N., Li W., Su W., Xu J., Bounsanga J., Ruiz-Negrón B., Lauren E., Licari F.W. (2019). Application of Machine Learning for Diagnostic Prediction of Root Caries. Gerodontology.

[154] Javed S., Zakirulla M., Baig R.U., Asif S.M., Meer A.B. (2020). Development of Artificial Neural Network Model for Prediction of Post-Streptococcus Mutans in Dental Caries. Comput. Methods Programs Biomed..

[155] Geetha V., Aprameya K.S., Hinduja D.M. (2020). Dental Caries Diagnosis in Digital Radiographs Using Back-Propagation Neural Network. Health Inf. Sci. Syst..

[156] Bayraktar Y., Ayan E. (2022). Diagnosis of Interproximal Caries Lesions with Deep Convolutional Neural Network in Digital Bitewing Radiographs. Clin. Oral Investig..

[157] Holtkamp A., Elhennawy K., Cejudo Grano de Oro J.E., Krois J., Paris S., Schwendicke F. (2021). Generalizability of Deep Learning Models for Caries Detection in Near-Infrared Light Transillumination Images. J. Clin. Med..

[158] Abdalla-Aslan R., Yeshua T., Kabla D., Leichter I., Nadler C. (2020). An Artificial Intelligence System Using Machine-Learning for Automatic Detection and Classification of Dental Restorations in Panoramic Radiography. Oral Surg. Oral Med. Oral Pathol. Oral Radiol..

[159] Krois J., Ekert T., Meinhold L., Golla T., Kharbot B., Wittemeier A., Dörfer C., Schwendicke F. (2019). Deep Learning for the Radiographic Detection of Periodontal Bone Loss. Sci. Rep..

[160] Kim E.-H., Kim S., Kim H.-J., Jeong H.-O., Lee J., Jang J., Joo J.-Y., Shin Y., Kang J., Park A.K. (2020). Prediction of Chronic Periodontitis Severity Using Machine Learning Models Based On Salivary Bacterial Copy Number. Front. Cell Infect. Microbiol..

[161] Huang W., Wu J., Mao Y., Zhu S., Huang G.F., Petritis B., Huang R.-P. (2020). Developing a Periodontal Disease Antibody Array for the Prediction of Severe Periodontal Disease Using Machine Learning Classifiers. J. Periodontol..

[162] Lee J.-H., Kim D.-H., Jeong S.-N., Choi S.-H. (2018). Diagnosis and Prediction of Periodontally Compromised Teeth Using a Deep Learning-Based Convolutional Neural Network Algorithm. J. Periodontal. Implant. Sci..

[163] Yauney G., Rana A., Wong L., Javia P., Muftu A. (2019). Automated Process Incorporating Machine Learning Segmentation and Correlation of Oral Diseases with Systemic Health. Proceedings of the 2019 41st Annual International Conference of the IEEE Engineering in Medicine & Biology Society (EMBC).

[164] Troiano G., Nibali L., Petsos H., Eickholz P., Saleh M.H.A., Santamaria P., Jian J., Shi S., Meng H., Zhurakivska K. (2023). Development and International Validation of Logistic Regression and Machine-Learning Models for the Prediction of 10-Year Molar Loss. J. Clin. Periodontol..

[165] Papantonopoulos G., Takahashi K., Bountis T., Loos B.G. (2014). Artificial Neural Networks for the Diagnosis of Aggressive Periodontitis Trained by Immunologic Parameters. PLoS ONE.

[166] Ozden F.O., Özgönenel O., Özden B., Aydogdu A. (2015). Diagnosis of Periodontal Diseases Using Different Classification Algorithms: A Preliminary Study. Niger. J. Clin. Pr..

[167] Alalharith D.M., Alharthi H.M., Alghamdi W.M., Alsenbel Y.M., Aslam N., Khan I.U., Shahin S.Y., Dianišková S., Alhareky M.S., Barouch K.K. (2020). A Deep Learning-Based Approach for the Detection of Early Signs of Gingivitis in Orthodontic Patients Using Faster Region-Based Convolutional Neural Networks. Int. J. Environ. Res. Public Health.

[168] Danks R.P., Bano S., Orishko A., Tan H.J., Moreno Sancho F., D’Aiuto F., Stoyanov D. (2021). Automating Periodontal Bone Loss Measurement via Dental Landmark Localisation. Int. J. Comput. Assist. Radiol. Surg..

[169] Ning W., Acharya A., Sun Z., Ogbuehi A.C., Li C., Hua S., Ou Q., Zeng M., Liu X., Deng Y. (2021). Deep Learning Reveals Key Immunosuppression Genes and Distinct Immunotypes in Periodontitis. Front. Genet..

[170] Wang C.-W., Hao Y., Di Gianfilippo R., Sugai J., Li J., Gong W., Kornman K.S., Wang H.-L., Kamada N., Xie Y. (2021). Machine Learning-Assisted Immune Profiling Stratifies Peri-Implantitis Patients with Unique Microbial Colonization and Clinical Outcomes. Theranostics.

[171] Li W., Liang Y., Zhang X., Liu C., He L., Miao L., Sun W. (2021). A Deep Learning Approach to Automatic Gingivitis Screening Based on Classification and Localization in RGB Photos. Sci. Rep..

[172] Revilla-León M., Gómez-Polo M., Vyas S., Barmak B.A., Galluci G.O., Att W., Krishnamurthy V.R. (2023). Artificial Intelligence Applications in Implant Dentistry: A Systematic Review. J. Prosthet. Dent..

[173] Lee S., Gantes B., Riggs M., Crigger M. (2007). Bone Density Assessments of Dental Implant Sites: 3. Bone Quality Evaluation during Osteotomy and Implant Placement. Int. J. Oral Maxillofac. Implant..

[174] Kernen F., Kramer J., Wanner L., Wismeijer D., Nelson K., Flügge T. (2020). A Review of Virtual Planning Software for Guided Implant Surgery—Data Import and Visualization, Drill Guide Design and Manufacturing. BMC Oral Health.

[175] Sadighpour L., Rezaei S., Paknejad M., Jafary F., Aslani P. (2014). The Application of an Artificial Neural Network to Support Decision Making in Edentulous Maxillary Implant Prostheses. J. Res. Pract. Dent..

[176] Lerner H., Mouhyi J., Admakin O., Mangano F. (2020). Artificial Intelligence in Fixed Implant Prosthodontics: A Retrospective Study of 106 Implant-Supported Monolithic Zirconia Crowns Inserted in the Posterior Jaws of 90 Patients. BMC Oral Health.

[177] Kurt Bayrakdar S., Orhan K., Bayrakdar I.S., Bilgir E., Ezhov M., Gusarev M., Shumilov E. (2021). A Deep Learning Approach for Dental Implant Planning in Cone-Beam Computed Tomography Images. BMC Med. Imaging.

[178] Lee D.-W., Kim S.-Y., Jeong S.-N., Lee J.-H. (2021). Artificial Intelligence in Fractured Dental Implant Detection and Classification: Evaluation Using Dataset from Two Dental Hospitals. Diagnostics.

[179] Thurzo A., Urbanová W., Novák B., Czako L., Siebert T., Stano P., Mareková S., Fountoulaki G., Kosnáčová H., Varga I. (2022). Where Is the Artificial Intelligence Applied in Dentistry? Systematic Review and Literature Analysis. Healthcare.

[180] Ding H., Wu J., Zhao W., Matinlinna J.P., Burrow M.F., Tsoi J.K.H. (2023). Artificial Intelligence in Dentistry—A Review. Front. Dent. Med..

[181] Junaid N., Khan N., Ahmed N., Abbasi M.S., Das G., Maqsood A., Ahmed A.R., Marya A., Alam M.K., Heboyan A. (2022). Development, Application, and Performance of Artificial Intelligence in Cephalometric Landmark Identification and Diagnosis: A Systematic Review. Healthcare.

[182] Thanathornwong B. (2018). Bayesian-Based Decision Support System for Assessing the Needs for Orthodontic Treatment. Healthc. Inf. Res..

[183] Xie X., Wang L., Wang A. (2010). Artificial Neural Network Modeling for Deciding If Extractions Are Necessary Prior to Orthodontic Treatment. Angle Orthod..

[184] Jung S.-K., Kim T.-W. (2016). New Approach for the Diagnosis of Extractions with Neural Network Machine Learning. Am. J. Orthod. Dentofac. Orthop..

[185] Choi H.-I., Jung S.-K., Baek S.-H., Lim W.H., Ahn S.-J., Yang I.-H., Kim T.-W. (2019). Artificial Intelligent Model With Neural Network Machine Learning for the Diagnosis of Orthognathic Surgery. J. Craniofac. Surg..

[186] Yu H.J., Cho S.R., Kim M.J., Kim W.H., Kim J.W., Choi J. (2020). Automated Skeletal Classification with Lateral Cephalometry Based on Artificial Intelligence. J. Dent. Res..

[187] Kök H., Acilar A.M., İzgi M.S. (2019). Usage and Comparison of Artificial Intelligence Algorithms for Determination of Growth and Development by Cervical Vertebrae Stages in Orthodontics. Prog. Orthod..

[188] Bianchi J., de Oliveira Ruellas A.C., Gonçalves J.R., Paniagua B., Prieto J.C., Styner M., Li T., Zhu H., Sugai J., Giannobile W. (2020). Osteoarthritis of the Temporomandibular Joint Can Be Diagnosed Earlier Using Biomarkers and Machine Learning. Sci. Rep..

[189] Kök H., Izgi M.S., Acilar A.M. (2021). Determination of Growth and Development Periods in Orthodontics with Artificial Neural Network. Orthod. Craniofac. Res..

[190] Aubreville M., Knipfer C., Oetter N., Jaremenko C., Rodner E., Denzler J., Bohr C., Neumann H., Stelzle F., Maier A. (2017). Automatic Classification of Cancerous Tissue in Laserendomicroscopy Images of the Oral Cavity Using Deep Learning. Sci. Rep..

[191] Hung M., Park J., Hon E.S., Bounsanga J., Moazzami S., Ruiz-Negrón B., Wang D. (2020). Artificial Intelligence in Dentistry: Harnessing Big Data to Predict Oral Cancer Survival. World J. Clin. Oncol..

[192] Brickley M.R., Shepherd J.P. (1996). Performance of a Neural Network Trained to Make Third-Molar Treatment-Planning Decisions. Med. Decis. Mak..

[193] Zhang W., Li J., Li Z.-B., Li Z. (2018). Predicting Postoperative Facial Swelling Following Impacted Mandibular Third Molars Extraction by Using Artificial Neural Networks Evaluation. Sci. Rep..

[194] Poedjiastoeti W., Suebnukarn S. (2018). Application of Convolutional Neural Network in the Diagnosis of Jaw Tumors. Healthcare Inf. Res..

[195] Murata M., Ariji Y., Ohashi Y., Kawai T., Fukuda M., Funakoshi T., Kise Y., Nozawa M., Katsumata A., Fujita H. (2019). Deep-Learning Classification Using Convolutional Neural Network for Evaluation of Maxillary Sinusitis on Panoramic Radiography. Oral Radiol..

[196] Endres M.G., Hillen F., Salloumis M., Sedaghat A.R., Niehues S.M., Quatela O., Hanken H., Smeets R., Beck-Broichsitter B., Rendenbach C. (2020). Development of a Deep Learning Algorithm for Periapical Disease Detection in Dental Radiographs. Diagnostics.

[197] Yang H., Jo E., Kim H.J., Cha I.-H., Jung Y.-S., Nam W., Kim J.-Y., Kim J.-K., Kim Y.H., Oh T.G. (2020). Deep Learning for Automated Detection of Cyst and Tumors of the Jaw in Panoramic Radiographs. J. Clin. Med..

[198] Johari M., Esmaeili F., Andalib A., Garjani S., Saberkari H. (2017). Detection of Vertical Root Fractures in Intact and Endodontically Treated Premolar Teeth by Designing a Probabilistic Neural Network: An Ex Vivo Study. Dentomaxillofac. Radiol..

[199] Saghiri M.A., Asgar K., Boukani K.K., Lotfi M., Aghili H., Delvarani A., Karamifar K., Saghiri A.M., Mehrvarzfar P., Garcia-Godoy F. (2012). A New Approach for Locating the Minor Apical Foramen Using an Artificial Neural Network. Int. Endod. J..

[200] Kositbowornchai S., Plermkamon S., Tangkosol T. (2013). Performance of an Artificial Neural Network for Vertical Root Fracture Detection: An Ex Vivo Study. Dent. Traumatol..

[201] Fukuda M., Inamoto K., Shibata N., Ariji Y., Yanashita Y., Kutsuna S., Nakata K., Katsumata A., Fujita H., Ariji E. (2020). Evaluation of an Artificial Intelligence System for Detecting Vertical Root Fracture on Panoramic Radiography. Oral Radiol..

[202] Orhan K., Bayrakdar I.S., Ezhov M., Kravtsov A., Özyürek T. (2020). Evaluation of Artificial Intelligence for Detecting Periapical Pathosis on Cone-Beam Computed Tomography Scans. Int. Endod. J..

[203] Mirbabaie M., Stieglitz S., Frick N.R.J. (2021). Artificial Intelligence in Disease Diagnostics: A Critical Review and Classification on the Current State of Research Guiding Future Direction. Health Technol..

[204] Mori Y., Kudo S.-E., East J.E., Rastogi A., Bretthauer M., Misawa M., Sekiguchi M., Matsuda T., Saito Y., Ikematsu H. (2020). Cost Savings in Colonoscopy with Artificial Intelligence-Aided Polyp Diagnosis: An Add-on Analysis of a Clinical Trial (with Video). Gastrointest. Endosc..

[205] Lee D., Yoon S.N. (2021). Application of Artificial Intelligence-Based Technologies in the Healthcare Industry: Opportunities and Challenges. Int. J. Environ. Res. Public Health.

[206] Salcedo J., Rosales M., Kim J.S., Nuno D., Suen S.-C., Chang A.H. (2021). Cost-Effectiveness of Artificial Intelligence Monitoring for Active Tuberculosis Treatment: A Modeling Study. PLoS ONE.

[207] Wani S.U.D., Khan N.A., Thakur G., Gautam S.P., Ali M., Alam P., Alshehri S., Ghoneim M.M., Shakeel F. (2022). Utilization of Artificial Intelligence in Disease Prevention: Diagnosis, Treatment, and Implications for the Healthcare Workforce. Healthcare.

[208] Tang H., Huang H., Liu J., Zhu J., Gou F., Wu J. (2022). AI-Assisted Diagnosis and Decision-Making Method in Developing Countries for Osteosarcoma. Healthcare.

[209] Fatima A., Shafi I., Afzal H., Díez I.D.L.T., Lourdes D.R.-S.M., Breñosa J., Espinosa J.C.M., Ashraf I. (2022). Advancements in Dentistry with Artificial Intelligence: Current Clinical Applications and Future Perspectives. Healthcare.

[210] Chan K.S., Zary N. (2019). Applications and Challenges of Implementing Artificial Intelligence in Medical Education: Integrative Review. JMIR Med. Educ..

[211] Li D. (2019). 5G and Intelligence Medicine-How the next Generation of Wireless Technology Will Reconstruct Healthcare?. Precis. Clin. Med..

[212] Joyce D.W., Kormilitzin A., Smith K.A., Cipriani A. (2023). Explainable Artificial Intelligence for Mental Health through Transparency and Interpretability for Understandability. NPJ Digit. Med..

[213] Goldhahn J., Rampton V., Spinas G.A. (2018). Could Artificial Intelligence Make Doctors Obsolete?. BMJ.

[214] Kumar P., Chauhan S., Awasthi L.K. (2023). Artificial Intelligence in Healthcare: Review, Ethics, Trust Challenges & Future Research Directions. Eng. Appl. Artif. Intell..

[215] Shaheen M.Y. (2021). Applications of Artificial Intelligence (AI) in Healthcare: A Review. Sci. Open.

[216] Celi L.A., Cellini J., Charpignon M.-L., Dee E.C., Dernoncourt F., Eber R., Mitchell W.G., Moukheiber L., Schirmer J., Situ J. (2022). Sources of Bias in Artificial Intelligence That Perpetuate Healthcare Disparities-A Global Review. PLOS Digit. Health.

[217] Abdullah Y.I., Schuman J.S., Shabsigh R., Caplan A., Al-Aswad L.A. (2021). Ethics of Artificial Intelligence in Medicine and Ophthalmology. Asia Pac. J. Ophthalmol..

[218] Reddy S., Allan S., Coghlan S., Cooper P. (2020). A Governance Model for the Application of AI in Health Care. J. Am. Med. Inf. Assoc..

[219] Rudin C., Radin J. (2019). Why Are We Using Black Box Models in AI When We Don’t Need To? A Lesson From An Explainable AI Competition. Harv. Data Sci. Rev..

[220] Ying X. (2019). An Overview of Overfitting and Its Solutions. J. Phys. Conf. Ser..

[221] Mashar M., Chawla S., Chen F., Lubwama B., Patel K., Kelshiker M.A., Bachtiger P., Peters N.S. (2023). Artificial Intelligence Algorithms in Health Care: Is the Current Food and Drug Administration Regulation Sufficient?. JMIR AI.

[222] Kermany D.S., Goldbaum M., Cai W., Valentim C.C.S., Liang H., Baxter S.L., McKeown A., Yang G., Wu X., Yan F. (2018). Identifying Medical Diagnoses and Treatable Diseases by Image-Based Deep Learning. Cell.

[223] Rosen J.M., Kun L., Mosher R.E., Grigg E., Merrell R.C., Macedonia C., Klaudt-Moreau J., Price-Smith A., Geiling J. (2016). Cybercare 2.0: Meeting the Challenge of the Global Burden of Disease in 2030. Health Technol..

[224] Houssami N., Kirkpatrick-Jones G., Noguchi N., Lee C.I. (2019). Artificial Intelligence (AI) for the Early Detection of Breast Cancer: A Scoping Review to Assess AI’s Potential in Breast Screening Practice. Expert Rev. Med. Devices.

[225] Labovitz D.L., Shafner L., Reyes Gil M., Virmani D., Hanina A. (2017). Using Artificial Intelligence to Reduce the Risk of Nonadherence in Patients on Anticoagulation Therapy. Stroke.

